# A scoping review on the health effects of smoke haze from vegetation and peatland fires in Southeast Asia: Issues with study approaches and interpretation

**DOI:** 10.1371/journal.pone.0274433

**Published:** 2022-09-15

**Authors:** Vera Ling Hui Phung, Attica Uttajug, Kayo Ueda, Nina Yulianti, Mohd Talib Latif, Daisuke Naito

**Affiliations:** 1 Center for Climate Change Adaptation, National Institute for Environmental Studies (NIES), Tsukuba, Ibaraki, Japan; 2 Department of Hygiene, Graduate School of Medicine, Hokkaido University, Sapporo, Hokkaido, Japan; 3 Department of Environmental Engineering, Graduate School of Engineering, Kyoto University, Kyoto, Kyoto, Japan; 4 Graduate School of Global Environmental Studies, Kyoto University, Kyoto, Kyoto, Japan; 5 Department of Agronomy, Faculty of Agriculture, Universitas Palangka Raya, Palangka Raya, Kalimantan Tengah, Indonesia; 6 Graduate Program of Environmental Science, Universitas Palangka Raya, Palangka Raya, Kalimantan Tengah, Indonesia; 7 Department of Earth Sciences and Environment, Faculty of Science and Technology, Universiti Kebangsaan Malaysia, Bangi, Selangor, Malaysia; 8 Graduate School of Agriculture, Kyoto University, Kyoto, Kyoto, Japan; 9 Center for International Forestry Research (CIFOR), Bogor, Jawa Barat, Indonesia; Universiti Teknologi Malaysia, MALAYSIA

## Abstract

Smoke haze due to vegetation and peatland fires in Southeast Asia is a serious public health concern. Several approaches have been applied in previous studies; however, the concepts and interpretations of these approaches are poorly understood. In this scoping review, we addressed issues related to the application of epidemiology (EPI), health burden estimation (HBE), and health risk assessment (HRA) approaches, and discussed the interpretation of findings, and current research gaps. Most studies reported an air quality index exceeding the ‘unhealthy’ level, especially during smoke haze periods. Although smoke haze is a regional issue in Southeast Asia, studies on its related health effects have only been reported from several countries in the region. Each approach revealed increased health effects in a distinct manner: EPI studies reported excess mortality and morbidity during smoke haze compared to non-smoke haze periods; HBE studies estimated approximately 100,000 deaths attributable to smoke haze in the entire Southeast Asia considering all-cause mortality and all age groups, which ranged from 1,064–260,000 for specified mortality cause, age group, study area, and study period; HRA studies quantified potential lifetime cancer and non-cancer risks due to exposure to smoke-related chemicals. Currently, there is a lack of interconnection between these three approaches. The EPI approach requires extensive effort to investigate lifetime health effects, whereas the HRA approach needs to clarify the assumptions in exposure assessments to estimate lifetime health risks. The HBE approach allows the presentation of health impact in different scenarios, however, the risk functions used are derived from EPI studies from other regions. Two recent studies applied a combination of the EPI and HBE approaches to address uncertainty issues due to the selection of risk functions. In conclusion, all approaches revealed potential health risks due to smoke haze. Nonetheless, future studies should consider comparable exposure assessments to allow the integration of the three approaches.

## 1. Introduction

Vegetation and peatland fires are gaining global attention owing to their increasing frequency and intensity. These events have been linked to climate change [1–3], as well as climatic [4] and anthropogenic factors [5–7]. Vegetation fires [8, 9] include natural wildfires and prescribed fires for socioeconomic purposes [10]. Meanwhile, peatland fires includes vegetation and the underlying peat layer [11], which are of high concern in equatorial areas with large organic (histosol) and peat soil volumes [12–15]. Both natural climatic factors [16] and prescribed fires [17] are important for balancing ecosystem mechanisms and land management. However, excessive and uncontrollable fires due to climate change have tremendous negative impacts on ecosystems [18] and human health [19, 20].

Vegetation and peatland fires in Southeast Asia are predominantly attributed to prescribed burning activities for economic and land use change purposes [21–23]. Moreover, dry weather conditions induced by the El Niño-Southern Oscillation or a positive Indian Ocean Dipole event [23, 24] intensify fires in the region. Generally, fire occurrences in Southeast Asia are classified into two main areas [7, 23]: mainland areas (Thailand, Myanmar, Laos, Vietnam, and Cambodia) and maritime areas (Malaysia, Brunei, Indonesia, Singapore, and the Philippines). The types and sources of fires are heterogeneous among countries in these areas. Indonesia and Malaysia have marked annual vegetation and peatland fire incidence [25, 26]. Countries located downwind of fire sources are affected by transboundary haze issues during the southwest monsoon season [27–29], in addition to fire and air pollutants from local sources [23, 29, 30]. The mainland is mostly affected by agricultural burning in the northern part of the area [23, 31, 32]. The complexity of haze occurrence across regions increases the challenges in assessing associated health risks.

Smoke released during vegetation and peatland fires contains a complex mixture of chemicals that are harmful to human health [33, 34]. These include particulate matter (PM) (e.g., PM10 and PM2.5) and its chemical constituents (e.g., elemental carbon, ionic species, elemental species, organic carbon), inorganic gases (e.g., carbon monoxide, ozone), hydrocarbons (e.g., polycyclic aromatic hydrocarbons (PAHs)), oxygenated organics (e.g., catechols, quinones), chlorinated organics (e.g., dioxin), and free radicals. Accumulating epidemiological evidence indicates the global health effects of fire smoke [35–37]. Several reviews on this topic have been published, including two that focused on Southeast Asian studies [38, 39]. These studies employed various approaches with different measures of health effects, namely (i) epidemiology (EPI), (ii) health burden estimation (HBE), and (iii) health risk assessment (HRA). The EPI approach is used to infer a causal association and allows quantification of the exposure-response relationship. The HBE approach is used to quantify the attributable health burden (using the exposure-response function derived from EPI studies) over an exposure at an average concentration of pollutant [19] or preventable mortality considering different scenarios [40, 41]. The HRA approach is *the process to estimate the nature and probability of adverse health effects in humans who may be exposed to chemicals in contaminated environmental media*, *now or in the future* [42].

Previous reviews have shown comprehensive literature on smoke haze-related health effects, but have not clearly addressed the differences among the three different approaches [38, 39]. Understanding the basic concepts and interpretation of findings of each approach is important since the results can be used to communicate health risks to the public and subsequently facilitate policy decisions. In this study, we performed a scoping review to summarize the trends of EPI, HBE, and HRA studies in Southeast Asia over the past few decades to clarify health effects, quantify exposure, interpret findings, as well as assess the underlying assumptions, strengths and limitations, and future challenges.

## 2. Methods

We conducted a literature search using online search engines, including PubMed, Scopus, and Web of Science, for scientific articles on vegetation fires and human health, published between 1990 and 2022. The general search terms related to vegetation and peatland fires or smoke haze events, human health, and Southeast Asia are shown in Table 1. Detailed search terms for each search engine are listed in S1 Table. Only full-text original or research articles on smoke haze and human health that were reported in studies conducted in Southeast Asia were included. Descriptive studies were also included if the haze episodes were explicitly mentioned. Gray literature was not considered in this study. Articles that focused on indoor exposure, occupational health, non-health-related issues, review articles, protocol papers, experimental study articles, letters, editorials, and commentaries were excluded. The results of this study were reported following the Preferred Reporting Items for Systematic reviews and Meta-Analyses extension for Scoping Reviews (PRISMA-ScR) guidelines [43] (S1 Checklist).

**Table 1:** Search terms by category.

Category	Search terms
Smoke haze and fire events	“forest fire” OR “peatland fire” OR “wildfire” OR “prescribed fire” OR “vegetation fire” OR “landscape fire” OR “agricultural burning” OR “transboundary haze” OR “smoke haze” OR “biomass burning” OR “bushfire” OR “haze”
Health	(“health” OR “mortality” OR “morbidity” OR “hospital admission” OR “emergency visit” OR “out-patient” OR “emergency ambulance dispatch*” OR “health risk assessment” OR “symptom” OR “respiratory” OR “cardiovascular” OR “cancer” OR “clinic visit*” OR “public health” OR “health risk*” OR “health impact” OR “mental health” OR “psychological” OR “death*” OR “asthma” OR “birth*” OR “low birth weight”) AND (“human” OR “epidemiol*”)
Study area	“Southeast Asia” OR “ASEAN” OR “Asia” OR “Malaysia” OR “Thailand” OR “Indonesia” OR “Laos” OR “Myanmar” OR “Cambodia” OR “Vietnam” OR “Singapore” OR “Brunei” OR “Philippines”
Time frame	“Jan/01/1990” to “Feb/28/2022”

Three authors (VP, AU, and KU) performed initial screening of the articles based on the title and abstract. With three equally distributed sets of articles, two of the three authors screened the same set of articles simultaneously. Any disagreement between the two was resolved through a discussion with the third author. References from full-text articles were manually searched. After identifying eligible articles, full-text articles were reviewed, and the data were extracted according to three approaches (EPI, HBE, and HRA).

The extracted data included the following: study approach, name of the first author, publication year, study area (country and area), study period, health endpoint analyzed, exposure assessment (pollutant of interest, levels of pollutants, exposure indicator of haze), measures of health outcomes, and results.

## 3. Results

### 3.1 Study selection

**Fig 1:**
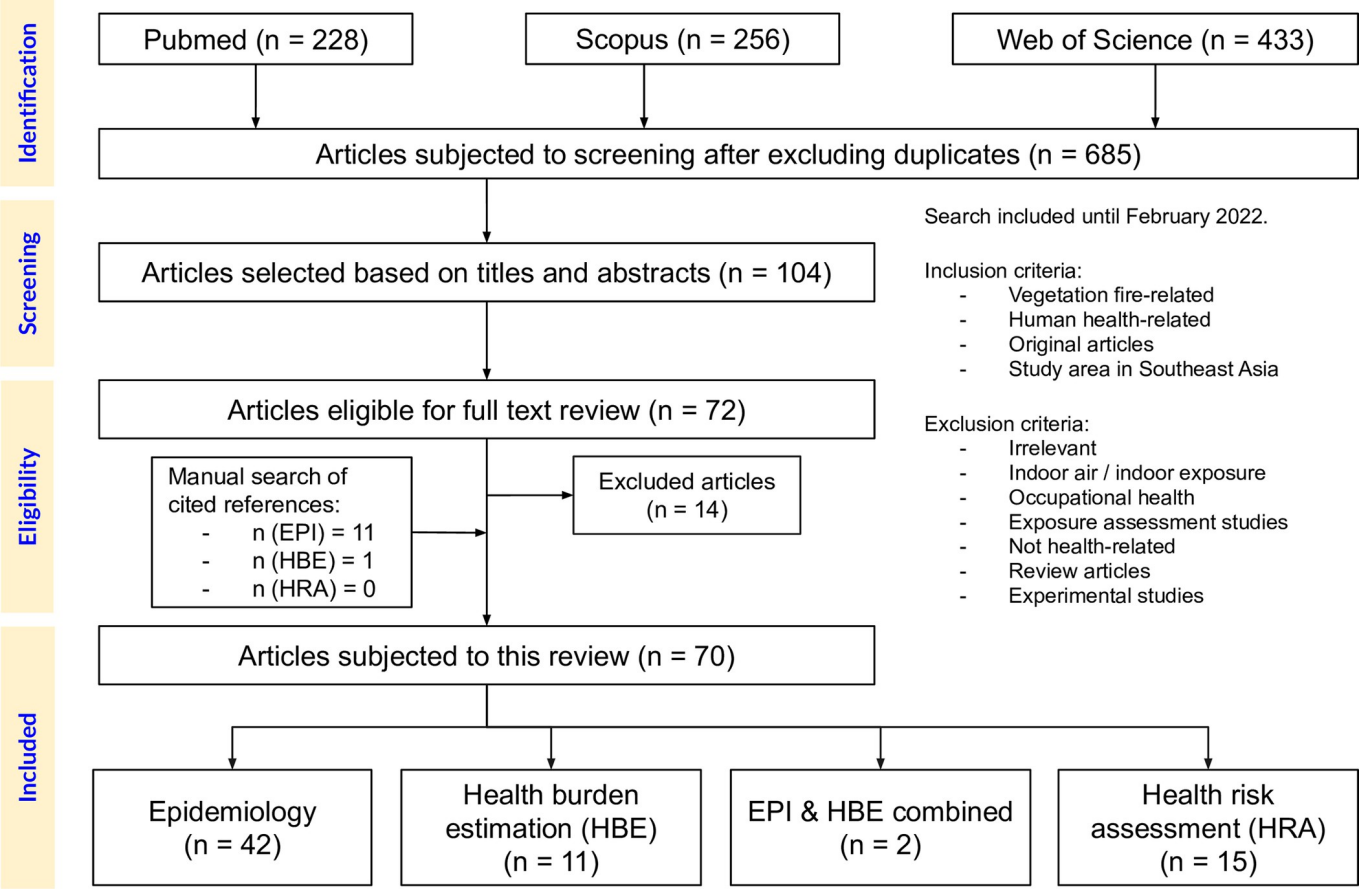
Flowchart of review process.

Fig 1 illustrates the selection process for this review. A total of 685 articles were identified. After de-duplicating the articles, title and abstract were screened; this yielded 104 articles. Of these, 58 met the eligibility criteria. Twelve studies were included in this manual search. Finally, 70 studies were included in this review.

### 3.2 Characteristics of the three approaches

**Fig 2:**
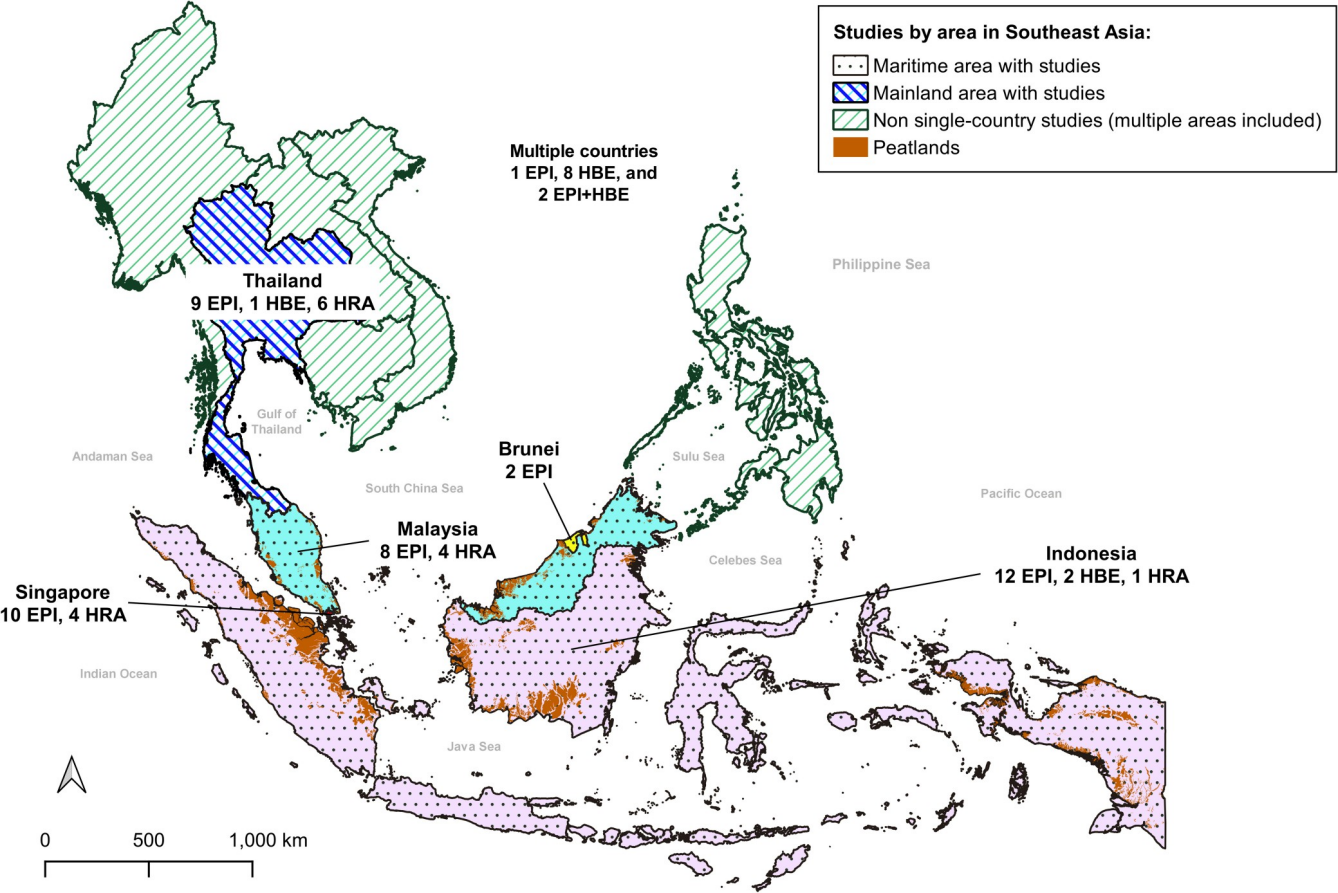
Map of countries where studies were conducted (Southeast Asia).

Table 2 summarizes the characteristics of each approach. Among the 70 studies, 42, 11, and 15 were EPI, HBE and HRA studies, respectively; two were both EPI and HBE. Forty-nine studies were conducted in the maritime area (Indonesia [41, 44–57], Malaysia [29, 58–68], Singapore [69–82], Brunei [83, 84], multiple countries in maritime area [40, 85–89]), 17 in the mainland area (Thailand [32, 90–104], multiple countries in mainland area [105]); and 4 in multiple countries in the entire Southeast Asia [19, 106–108] (Fig 2). The breakdown of studies by country is as follows: 41 EPI [32, 44–46, 49–58, 61–67, 69, 70, 75–84, 90, 97–103], three HBE [41, 47, 104], and 15 HRA [29, 48, 59, 60, 68, 71–74, 91–96] studies were conducted in a single country (Indonesia, Malaysia, Singapore, Brunei, and Thailand); whereas one EPI study [85], eight HBE [19, 40, 86–89, 105, 106] and two EPI- and HBE-combined studies were conducted in multiple countries [107, 108]. Except for four EPI studies [45, 53, 55, 102], majority of the studies examined the health effects of short-term haze exposure, focusing on the daily variation of air pollutants. Three HBE studies estimated the mortality attributed to smoke haze using short-term (daily) and long-term (annual) air pollution levels [19, 86, 106]. Eight HBE studies [40, 41, 47, 87–89, 104, 105] and 15 HRA studies [29, 48, 59, 60, 68, 71–74, 91–96] examined the health effects of long-term exposure. Ten EPI studies [32, 52, 56, 58, 63, 66, 67, 82, 101, 102], 11 HBE studies [19, 40, 41, 47, 86–89, 104–106], and two EPI- and HBE-combined studies [107, 108] assessed mortality as a health endpoint; 35 EPI studies assessed morbidity [32, 44–46, 49–51, 53–57, 61, 62, 64–66, 69, 70, 75–81, 83–85, 90, 97–100, 102]; whereas all HRA studies [29, 48, 59, 60, 68, 71–74, 91–96] assessed potential cancer and non-cancer risks, which could not be clearly distinguished as mortality or morbidity.

**Table 2:** Summary of studies on the health effects of smoke haze in Southeast Asia.

Approach	Author (Year)	Location	Study period	Exposure time^a^	Health endpoint	Pollutant	Exposure assessment and indicators of exposure	Exposure concentration^b^	Air Quality Index^c^
**EPI**	Brauer and Hisham-Hashim (1998) [85]	Malaysia and Singapore	Aug–Sep 1997	S	Morbidity (respiratory)	NA	NA	NA	NA
**EPI**	Aditama (2000) [44]	Indonesia	Sep 1997–Jun 1998	S	Morbidity (respiratory)	NA	NA	NA	NA
**EPI**	Emmanuel (2000) [69]	Singapore	Aug–Nov 1997	S	Morbidity (respiratory)	PM10	Temporal comparison and binary indicator defined by PM10 >50 μg/m^3^	60–100 μg/m^3^ (The highest PSI recorded was 134)	(PSI: 134) Unhealthy
**EPI**	Tan et al. (2000) [70]	Singapore	Jun–Dec 1997	S	Morbidity (respiratory)	PM10, CO, O_3_, NO_2_, SO_2_	Haze: Sep 29–Oct 27, 1997 Post-haze: Nov 21–Dec 5, 1997	Daily mean PM10 (haze period) 125.4 μg/m^3^ (post-haze period) 40.0 μg/m^3^	(AQI_haze_: 86) Moderate (AQI_post-haze_: 37) Good
**EPI**	Odihi (2001) [83]	Muara and Temburong, Brunei Darussalam	Sep 1997–Jun 1998 and Jan–Jun 1997–Sep 1998	S	Morbidity (respiratory)	NA	Temporal comparison (Sep–Oct of 1997)	NA	NA
**EPI**	Kunii et al. (2002) [50]	Jambi, Indonesia	Sep 29, 1997, and Oct 7, 1997	S	Morbidity (symptoms)	NA	NA	Daily maximum PM10: 1,824 μg/m^3^	(AQI >500) Hazardous
**EPI**	Sastry (2002) [58]	Kuala Lumpur, Johor Bahru, Ipoh, Kuching, Penang, Malaysia	1996–1997	S	Mortality (all-cause)	PM10, visibility	Binary indicator defined by PM10 >210 μg/m^3^, or visibility <0.91 km	Daily mean PM10: 64.2 μg/m^3^ Daily mean visibility: 6.8 km Daily maximum PM10: 423.9 μg/m^3^	(AQI_mean_: 55) Moderate (AQI_max_: 299) Very Unhealthy
**EPI**	Anaman and Ibrahim (2003) [84]	Brunei-Muara district, Brunei Darussalam	Jan–Apr 1998	S	Morbidity (respiratory)	PSI	PSI	NA	NA
**EPI**	Frankenberg et al. (2005) [51]	Indonesia	1997	S	Morbidity (general)	TOMS aerosol index	Binary indicator defined by TOMS aerosol index	Maximum: 6	NA
**EPI**	Mott et al. (2005) [61]	Kuching, Malaysia	1995–1998	S	Morbidity (cardiorespiratory diseases)	NA	Temporal comparison (Aug–Oct 1997)	Daily maximum PM10 (on Sep 22, 1997): 852 μg/m^3^	(AQI >500) Hazardous
**EPI**	Jayachandran (2009) [52]	Indonesia	1997	S	Mortality (fetal, infant, and children)	TOMS aerosol index	TOMS aerosol index	Daily mean: 0.120 (Aug–Oct 1996): 0.048 (Aug–Oct 1997): 0.578	NA
**EPI**	Wiwatanadate and Liwsrisakun (2011) [90]	Chiang Mai, Thailand	Aug 15, 2005– Jun 30, 2006	S	Morbidity (respiratory)	PM2.5, PM10, O_3_, NO_2_, SO_2_	PM2.5, PM10, O_3_, NO_2_, SO_2_	Daily mean PM2.5: 43.8 μg/m^3^ PM10: 58.1 μg/m^3^ CO:1.09 ppm O_3_: 17.5 ppb NO_2_: 17.2 ppb SO_2_: 1.7 ppb Daily maximum PM2.5: 310 μg/m^3^ Daily maximum PM10: 335 μg/m^3^	(AQI_PM2.5-max_: 360) Hazardous
**EPI**	Ho, R.C. et al. (2014) [75]	Singapore	Jun 21–26, 2013	S	Morbidity (psychological)	NA	NA	NA	NA
**EPI**	Othman et al. (2014) [62]	Selangor, Malaysia	2005–2006, 2008–2009	S	Morbidity (respiratory)	API, PM10	Binary indicator defined by API	Daily mean PM10: (haze days) 168 μg/m^3^ (non-haze days) 51.7 μg/m^3^ Daily mean API: 54.6 (110 days with API beyond “unhealthy” level)	(AQI_haze-PM10_: 107) Unhealthy for Sensitive Groups (AQI_nonhaze-PM10_: 47) Good (API: 54.6) Upper-moderate
**EPI**	Sahani et al. (2014) [63]	Klang Valley, Malaysia	2000–2007	S	Mortality (respiratory, natural)	PM10	Binary indicator defined by PM10 >100 μg/m^3^	Daily mean: (overall) 55.5 μg/m^3^ (haze days) 134.5 μg/m^3^ (non-haze days) 53.1 μg/m^3^	(AQI_overall_: 51) Moderate (AQI_haze_: 90) Moderate (AQI_non-haze_: 49) Good
**EPI**	Yeo et al. (2014) [76]	Singapore	Jun 25, 2013–Jul 11, 2013	S	Morbidity (respiratory)	NA	NA	NA	NA
**EPI**	Pothirat et al. (2016) [97]	Chiang Mai, Thailand	Jan–Mar, 2006–2009	S	Morbidity (respiratory)	PM10	PM10	Daily median: 64.5 μg/m^3^	(AQI: 55) Moderate
**EPI**	Hassan et al. (2017) [64]	Kuala Lumpur, Malaysia	Jan 2010– Oct 2015	S	Morbidity (Lung cancer)	Visibility	Binary indicator defined by visibility <10 km	NA	NA
**EPI**	Kim et al. (2017) [53]	Indonesia	1993, 1997, 2000, 2007	L	Morbidity (respiratory)	TOMS aerosol index	TOMS aerosol index	NA	NA
**EPI**	Sheldon and Sankaran (2017) [77]	Center of Singapore, Singapore	2010–2016	S	Morbidity (respiratory)	PSI	PSI	Daily mean: 39 Daily maximum: 258	(PSI_max_: 258) Very unhealthy
**EPI**	Syam et al. (2017) [49]	Borneo and Sumatra, Indonesia	Oct 2015– Nov 2015	S	Morbidity (respiratory)	NA	Self-reported hours of smoke exposure	NA	NA
**EPI**	Ho, A.F.W. et al. (2018a) [78]	Singapore	2010–2015	S	Morbidity (cardiovascular)	PSI	Categorical and continuous indicator of PSI	Daily mean: 36 Daily maximum: 197.6	(PSI_mean_: 36) Good (PSI_max_: 197.6) Unhealthy
**EPI**	Ho, A.F.W. et al. (2018b) [79]	Singapore	2010–2015	S	Morbidity (cardiovascular)	PSI	Categorical and continuous indicator of PSI	Daily median: (Overall): 32.8 (PSI_good-period_): 29.5 (PSI_moderate-period_): 58.4 (PSI_unhealthy-period_): 130.3	(PSI_overall_: 32.8) Good During ‘unhealthy-PSI’ period, average PSI: 130.3
**EPI**	Ming et al. (2018) [65]	Klang Valley, Malaysia	2014–2015	S	Morbidity (respiratory)	Visibility	Binary indicator defined by visibility <10 km	NA	NA
**EPI**	Ho, A.F.W. et al. (2019) [80]	Singapore	2010–2015	S	Morbidity (cardiovascular)	PSI	Categorical and continuous indicator of PSI	Daily median: 32.8 41 days were identified in ‘unhealthy’ PSI (PSI≥101)	(PSI: 32.8) Good 41 days were identified in ‘unhealthy’ PSI (PSI≥101)
**EPI**	Pothirat et al. (2019) [98]	Chiang Dao district, Thailand	Mar and Aug 2016	S	Morbidity (respiratory)	NA	Temporal comparison (March)	Daily mean PM10: (low-PM10 period) 29.2 μg/m^3^ (high-PM10 period) 120.4 μg/m^3^	(AQI_low-period_: 27) Good (AQI_high-period_: 83) Moderate
**EPI**	Suyanto et al. (2019) [54]	Pekanbaru, Indonesia	2015–2016	S	Morbidity (respiratory)	PM10 and AQI	Temporal comparison (2015)	AQI >300 on 9 Sep 2015 AQI <100 in 2016	(AQI >300) Hazardous
**EPI**	Tan-Soo and Pattanayak (2019) [55]	Sulawesi, Nusa Tenggara, Kalimantan, Sumatra, Indonesia	1997, 2000, 2007, 2014	L	Morbidity (nutrition)	TOMS aerosol index	Aerosol Index	Annual average aerosol index range: 0.1–0.3	NA
**EPI**	Aik et al. (2020) [81]	Singapore	2009–2018	S	Morbidity (acute conjunctivities)	PM2.5, PM10	Haze episode (details were not described)	Weekly mean PM2.5: 18.4 μg/m^3^ Weekly mean PM10: 30.0 μg/m^3^	NA
**EPI**	Ho A.F.W. et al. (2020) [82]	Singapore	2010–2015	S	Mortality (all-cause)	PSI	Categorical and continuous indicator of PSI	Daily median: (Overall): 32.8 (PSI_good-period_): 29.5 (PSI_moderate-period_): 58.4 (PSI_unhealthy-period_): 130.3	(PSI_overall_: 32.8) Good During ‘unhealthy-PSI’ period, average PSI: 130.3
**EPI**	Mueller et al. (2020) [99]	Upper north region, Thailand	2014–2017	S	Morbidity (respiratory)	PM10	PM10	Daily mean: (haze days) 74.6 μg/m^3^ (non-haze days) 26.0 μg/m^3^	(AQI_haze_: 60) Moderate (AQI_non-haze_: 24) Good
**EPI**	Ontawong et al. (2020) [100]	Pong District, Phayao Province, Thailand	4 years (Not specified)	L	Morbidity (respiratory)	NA	NA	NA	NA
**EPI**	Vajanapoom et al. (2020) [101]	Chiang Mai, Thailand	2002–2016	S	Mortality (all-cause)	PM10	PM10, NO_2_, SO_2_, O_3_, CO	Daily mean: (Period 1: before haze control) 54.3 μg/m^3^ (Period 2: haze control initiated) 42.2 μg/m^3^ (Period 3: haze control continued) 45.2 μg/m^3^	(AQI_Period-1_: 50) Good (AQI_Period-2_: 39) Good (AQI_Period-3_: 42) Good
**EPI**	Zaini et al. (2020) [56]	Riau, Pekanbaru, Indonesia	2015	S	Morbidity (respiratory)	NA	NA	NA	NA
**EPI**	Jaafar et al. (2021) [66]	Selangor, Malaysia	2012–2015	S	Morbidity (respiratory)	PM10	Binary indicator defined by PM10 ≥51 μg/m^3^	Daily maximum: 595.1 μg/m^3^ (July 2013)	(AQI: 491) Hazardous
**EPI**	Mueller et al. (2021) [102]	Thailand	Jan 1, 2015–Apr 30, 2018	L	Morbidity (birthweight)	PM10	Fire hotspots by satellite data	Mean (entire pregnancy): 39.7 μg/m^3^	NA
**EPI**	Pothirat et al. (2021) [103]	Chiang Mai, Thailand	2016–2018	S	Mortality (all-cause, cause-specific)	PM2.5, PM10	PM2.5, PM10	Daily median PM10: 39.5 μg/m^3^ Daily median PM2.5: 18.2 μg/m^3^	(AQI_PM10_: 36) Good (AQI_PM2.5_: 64) Moderate
**EPI**	Uttajug et al. (2021) [32]	Upper north Thailand (8 provinces), Thailand	2014–2018	S	Morbidity (respiratory)	PM10	PM10	Daily mean: (haze days) 122.9–165.1 μg/m^3^ (non-haze days) 18.0–30.4 μg/m^3^	(AQI_haze_: 106) Unhealthy for Sensitive Groups (AQI_non-haze_: 28) Good
**EPI**	Astuti et al. (2022) [57]	Palangka Raya, Central Kalimantan, Indonesia	Oct 2015	S	Morbidity (respiratory)	NA	Fire hotspots by satellite data	Daily maximum PM10: 775 μg/m^3^ (Mid-Oct)	(AQI >500) Hazardous
**EPI**	Jalaludin et al. (2022) [45]	Indonesia	2000, 2007/2008, 2014/2015	L	Morbidity (cognitive function)	PM2.5	NA	Annual mean: (fire-prone provinces) 9.7 μg/m^3^ (non fire-prone provinces) 12.7 μg/m^3^	NA
**EPI**	Phung et al. (2022) [67]	12 districts in Malaysia	2014–2016	S	Mortality	PM10	Binary indicator defined by PM10 >50 μg/m^3^, >75μg/m^3^, >100μg/m^3^, and >150μg/m^3^, and duration of occurrence	Daily mean (averaged for 12 districts): 52.8 μg/m^3^ 231 days identified with PM10 >150 μg/m^3^	(AQI: 48) Good 231 days were identified with ‘unhealthy’ air quality level (API ≥101)
**EPI**	Siregar et al. (2022) [46]	Sumatra, Indonesia	2007–2008	L	Morbidity (cardiovascular)	PM2.5	PM2.5	Annual mean: 14.43 μg/m^3^	NA
**HBE**	Johnston et al. (2012) [19]	Global and regional (Southeast Asia)	1997–2006	L/S	Mortality	PM2.5	PM2.5	Annual average: 1.8 μg/m^3^ Population-weighted annual average: 2.1 μg/m^3^	NA
**HBE**	Marlier et al. (2013) [106]	Southeast Asia	1997–2006	L/S	Mortality (cardiovascular)	PM2.5, O_3_	PM2.5, O_3_	Annual average fire-PM2.5: 8.3 μg/m^3^ (1997), 0.4 μg/m^3^ (2000) Annual average fire-O_3_: 8.0 ppb (1997), 1.4 ppb (2000)	NA
**HBE**	Crippa et al. (2016) [86]	Maritime Southeast Asia	Sep–Oct 2015	L/S	Mortality	PM2.5	PM2.5	Daily mean PM2.5: 45.12 μg/m^3^ Daily mean PM10: 155.28 μg/m^3^	(AQI_PM2.5_: 125, AQI_PM10_: 101) Unhealthy for Sensitive Groups
**HBE**	Koplitz et al. (2016) [87]	Maritime Southeast Asia	Sep–Oct, 2006 and 2015	L	Mortality	PM2.5	PM2.5	Seasonal (Jul–Oct) mean: (fire-related) 14–27 μg/m^3^ (non-fire PM2.5) 10–15 μg/m^3^	NA
**HBE**	Marlier et al. (2019) [40]	Maritime Southeast Asia	Projection for 2020–2029	L	Mortality	PM2.5	PM2.5	Seasonal mean population-weighted (Jul–Oct): (Indonesia) 6.6 μg/m^3^, (Malaysia) 5.5 μg/m^3^, (Singapore) 6.0 μg/m^3^	NA
**HBE**	Uda et al. (2019) [47]	Indonesia	2011–2015	L	Mortality	PM2.5	PM2.5	Annual mean fire-PM2.5: 26 μg/m^3^	NA
**HBE**	Bruni Zani et al. (2020) [88]	Maritime Southeast Asia	2005–2015	L	Mortality	PM2.5	PM2.5	Seasonal mean PM10: (Jun–Aug) 50.90 μg/m^3^ (Dec–Feb) 45.63 μg/m^3^	NA
**HBE**	Kiely et al. (2020) [89]	Maritime Southeast Asia	2004–2015	L	Mortality, Years of life lost (YLL), Disability-adjusted life years (DALY)	PM2.5	PM2.5	Annual average: 76 μg/m^3^ (Population-weighted: 27 μg/m^3^) Under peatland protection, annual average: 55 μg/m^3^ (Population-weighted: 20 μg/m^3^)	NA
**HBE**	Kiely et al. (2021) [41]	Indonesia	2004–2015	L	Mortality, DALY	PM2.5	PM2.5 reduction due to peatland restoration	In 2015, reduction of 28% (from 76 μg/m^3^ to 55 μg/m^3^) average PM2.5 emission, and 26% population-weighted PM2.5 (from 27 μg/m^3^ to 20 μg/m^3^)	NA
**HBE**	Punsompong et al. (2021) [104]	Thailand	2016	L	Mortality (stroke, ischemic heart disease, lung cancer, and COPD)	PM2.5	PM2.5	Annual mean PM2.5: (Central and Northeast region) 26–40 μg/m^3^ (North region) >40 μg/m^3^	NA
**HBE**	Reddington et al. (2021) [105]	Southern Asia (Mainland Southeast Asia (Cambodia, Laos, Myanmar, Thailand, and Vietnam), and Southeast China)	2003–2015	L	Mortality	PM2.5, Ozone	PM2.5, Ozone	NA	NA
**EPI- and HBE-combined**	Chen et al. (2021) [107]	Global (43 countries) (included Thailand and Philippines in Southeast Asia)	[Global] 200–2016; [Thailand] 2000–2008; [Philippines] 2006–2010	S	Mortality (all-cause, cardiovascular, respiratory)	PM2.5	PM2.5	Daily mean fire-PM2.5: 0.17–4.36 μg/m^3^ Daily maximum fire-PM2.5: 3.46–178 μg/m^3^	NA
**EPI- and HBE-combined**	Xue et al. (2021) [108]	Global (192 countries) (Southeast Asia: Indonesia, Myanmar, Vietnam, Cambodia, Philippines, Thailand, Laos, Malaysia, Singapore, Brunei)	2000–2014	L	Mortality	PM2.5	PM2.5	Monthly mean fire-PM2.5: 4.06 μg/m^3^	NA
**HRA**	Omar et al. (2006) [68]	Kuala Lumpur, Malaysia	Mar 22–Dec 12, 2001	L	Carcinogenic risk	PAH	PAH	NA	NA
**HRA**	Betha et al. (2013) [48]	Kalimantan, Indonesia	Sep 19–Oct 12, 2009	L	Carcinogenic and non-carcinogenic risks	Trace metal elements	Trace metal elements	Daily maximum PM2.5: 7,817 μg/m^3^	(AQI >500) Hazardous
**HRA**	Wiriya et al. (2013) [91]	Chiang Mai, Thailand	Apr 2010, Aug–Oct 2010, and Jan–Mar 2011	L	Carcinogenic risk	PAH	PAH	Seasonal mean PM10: (dry season 2010) 104.91 μg/m^3^ (wet season 2010) 13.28 μg/m^3^ (dry season 2011) 36.24 μg/m^3^	NA
**HRA**	Betha et al. (2014) [71]	Singapore	Jun 20–28, and Sep 12–Oct 2, 2013	L	Carcinogenic and non-carcinogenic risks	Trace metal elements	Trace metal elements	Daily PM2.5: (haze days) 54–329 μg/m^3^ (non-haze days) 11–21 μg/m^3^	(AQI_haze_: 147–379) Unhealthy for Sensitive Groups-Hazardous (AQI_non-haze_: 46–79) Good–Moderate
**HRA**	Pongpiachan et al. (2015) [92]	(9 provinces in upper north region) Thailand	Nov 2012–Mar 2013	L	Carcinogenic risk	PAH	PAH	NA	NA
**HRA**	Huang et al. (2016) [72]	Singapore	Jan–Sep 2014	L	Carcinogenic and non-carcinogenic risks	Trace metal elements	Trace metal elements	Daily mean PM2.5: (haze days) 61.2 μg/m^3^ (non-haze days) 22.0 μg/m^3^	(AQI_haze_: 154) Unhealthy (AQI_non-haze_: 72) Moderate
**HRA**	Khan et al. (2016) [59]	Bangi, Selangor, Malaysia	Jul–Sep 2013, and Jan–Feb 2014	L	Carcinogenic and non-carcinogenic risks	Trace metal elements and ionic species	Trace metal elements and ionic species	Daily mean PM2.5: 25.13 μg/m^3^	(AQI: 78) Moderate
**HRA**	Sulong et al. (2017) [29]	Kuala Lumpur, Malaysia	Jun 2015– Jan 2016	L	Carcinogenic and non-carcinogenic risks	Trace metal elements and ionic species	Trace metal elements and ionic species	Daily mean PM2.5: (pre-haze) 24.5 μg/m^3^ (haze) 72.3 μg/m^3^ (post-haze) 14.3 μg/m^3^	(AQI_pre-haze_: 77) Moderate (AQI_haze_: 160) Unhealthy (AQI_post-haze_: 56) Moderate
**HRA**	Urbancok et al. (2017) [73]	Singapore	May 2015– May 2016	L	Carcinogenic risk	PAH	PAH	Daily PM10: (haze days; Sep–Oct) 72–323 μg/m^3^ (non-haze days) 32–70 μg/m^3^	(AQI_haze_: 59–185) Moderate–Unhealthy (AQI_non-haze_: 30–58) Good–Moderate
**HRA**	Sharma and Balasubramanian (2018) [74]	Singapore	7 days in Oct 2015	L	Carcinogenic and non-carcinogenic risks	Trace metal elements	Trace metal elements	Daily mean PM2.5: (light-haze) 47 μg/m^3^ (moderate-haze) 101 μg/m^3^ (severe-haze) 134 μg/m^3^	(AQI_light-haze_: 129) Unhealthy for Sensitive Group (AQI_moderate-haze_: 174) Unhealthy (AQI_severe-haze_: 192) Unhealthy
**HRA**	Sulong et al. (2019) [60]	Kuala Lumpur, Malaysia	Jun 2015– May 2016	L	Carcinogenic risk	PAH	PAH	NA	NA
**HRA**	Pani et al. (2020) [93]	Chiang Mai, Thailand	19 Mar–11 May 2016	L	Carcinogenic and non-carcinogenic risks	Black carbon	Black carbon	Daily mean PM2.5: 68–71 μg/m^3^	(AQI: 157–159) Unhealthy
**HRA**	Thepnuan et al. (2020) [94]	Chiang Mai, Thailand	Feb 23–Apr 28, 2016	L	Carcinogenic risk	PAH	PAH	Daily mean PM2.5: 64.3 μg/m^3^	(AQI: 156) Unhealthy
**HRA**	Yabueng et al. (2020) [95]	Chiang Mai and Nan Provinces, Thailand	Mar–Apr, 2017–2018	L	Carcinogenic risk	PAH	PAH	Daily mean PM2.5: 37.93–41.86 μg/m^3^	(AQI: 107–117) Unhealthy for Sensitive Groups
**HRA**	Insian et al. (2022) [96]	Chiang Mai, Thailand	Mar-Jun & Nov, 2019	L	Carcinogenic risk	PAH	PAH	Seasonal PM during haze days: (urban area) 105.1 μg/m^3^ (rural area) 128.4 μg/m^3^	NA

EPI: epidemiological approach; HBE: health burden estimation approach; EPI- and HBE-combined: a design that combined EPI and HBE approaches in one study; HRA: health risk assessment approach; PSI: pollutant standard index; TOMS: total ozone mapping spectrometer; API: air pollutant index; PAH: polycyclic aromatic hydrocarbon; COPD: chronic obstructive pulmonary disorder.

^a^ Exposure time is indicated by “S” for short-term and “L” for long-term exposure.

^b^ Exposure concentration reported for the pollutant specified in the “Pollutant” column. Pollutants are otherwise specified if there is information on the concentrations of several types of pollutants.

^c^ Values denote air quality index (AQI) based on the US EPA calculation [109]. AQI is marked as not applicable “NA” under these circumstances: (i) value reported is not daily exposure; (ii) no exposure value reported, or country-specific national AQI (e.g., PSI (Singapore; Brunei Darussalam), API (Malaysia)) is reported; or (iii) only fire-originated pollutant concentration is reported. The PSI and API values from the original studies are listed here if reported in previous studies. The AQI values and indicators [109] are as follows: (i) 0≤AQI≤50 “Good”; (ii) 51≤AQI≤100 “Moderate”; (iii) 101≤AQI≤150 “Unhealthy for Sensitive Groups”; (iv) 151≤AQI≤200 “Unhealthy”; (v) 201≤AQI≤300 “Very Unhealthy”; and (vi) AQI≥301 “Hazardous”.

Data were obtained from the GADM maps and data [110]. Peatland information was reprinted from previous studies [111] under a CC by license, and with permission from Dr. Nina Yulianti, original copyright (2013, 2016) [112, 113].

**Fig 3:**
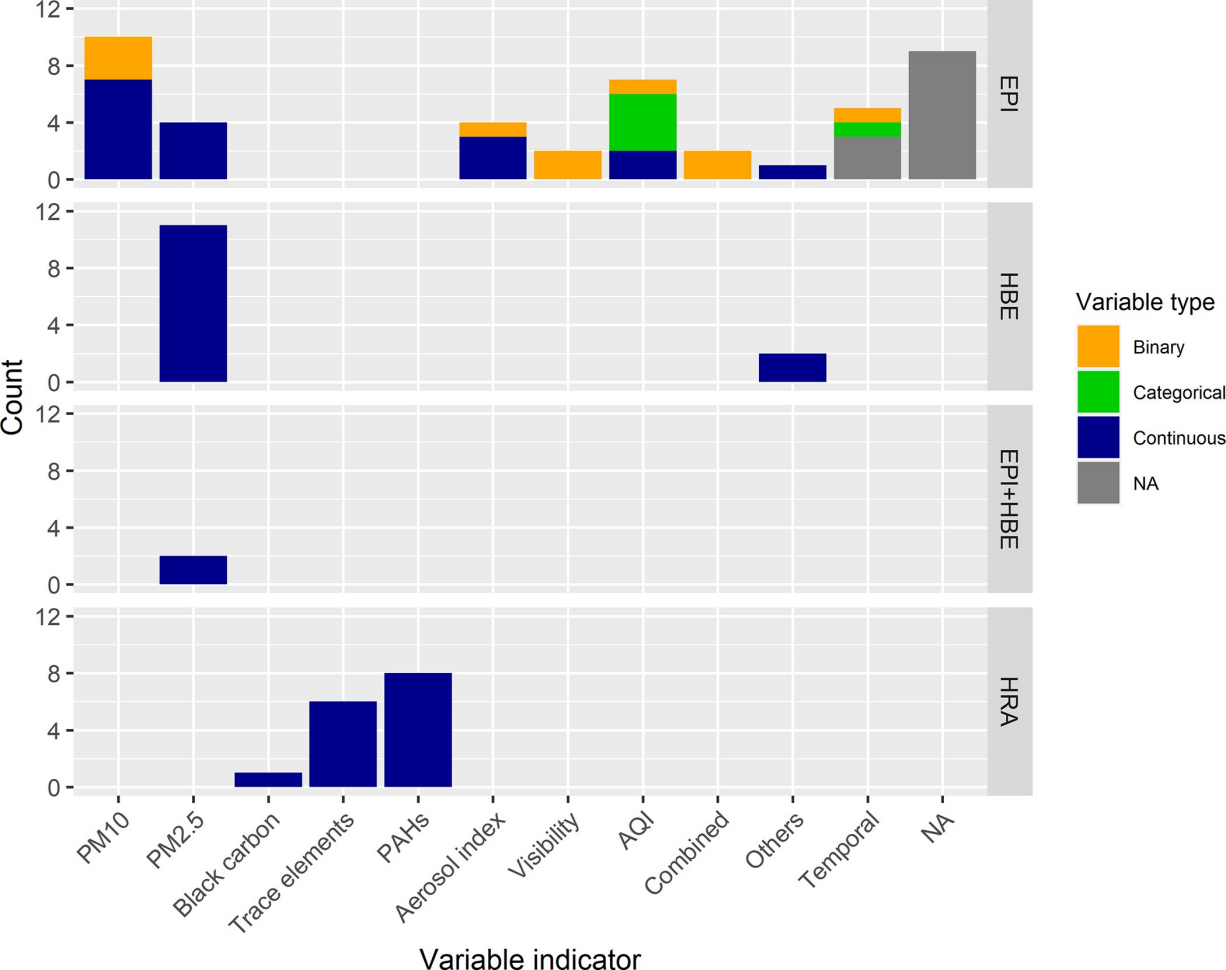
Exposure indicators used for each approach. AQI: air quality index (generally referred to as “AQI,” although different terms can be used (e.g., PSI (Pollutant Standard Index), API (Air Pollutant Index)); EPI + HBE: studies that applied a combination of EPI and HBE approaches; Combined exposure indicators were defined as a combination of pollutants and pollutant indicators; Others: pollutants other than those specified; NA: no specific variables are used.

EPI studies used various exposure indicators, including PM2.5, PM10, air quality indices (AQI, PSI, API), PM constituents, and the total ozone mapping spectrometer (TOMS) aerosol index (Fig 3). Among the 12 studies that did not use specific exposure indicators, seven described haze-related diseases [44, 49, 50, 56, 57, 76, 85], lung function [100], and symptoms with perceived PSI level [75], and three made a temporal comparison of health outcomes between haze and non-haze periods [61, 83, 98]. All HBE studies [19, 40, 41, 47, 86–89, 104] and all EPI- and HBE-combined studies [107, 108] used PM2.5 as the exposure indicator; whereas, two HBE studies [105, 106] used both PM2.5 and ozone as indicators. HRA studies used specific PM constituents such as PAHs [60, 68, 73, 91, 92, 94–96], trace metal elements [29, 48, 59, 71, 72, 74], and black carbon [93] as exposure indicators.

#### 3.2.1 Epidemiology approach (EPI)

Among the 42 epidemiological studies, 33 were conducted in the maritime area and nine were conducted in Thailand in the mainland area (Table 2, S2 Table). Studies from Indonesia, Singapore, Malaysia and Brunei Darussalam mainly focused on specific smoke haze episodes, whereas studies from Thailand were more focused on the health effects of seasonal haze due to burning for agricultural purposes in the northern mountainous areas [32, 90, 97–101, 103].

The 42 studies were classified into eight descriptive and 34 analytical studies examining the association between exposure and diseases (S2 Table). Descriptive studies reported the number of hospital visits owing to respiratory diseases [44, 85] and the prevalence of respiratory symptoms [49, 50, 56, 57, 76, 83] during fire episodes. Headache and eye irritation are the main non-respiratory symptoms frequently reported in Indonesia [49, 50, 56] and Brunei [83]. Only five studies examined the long-term effects of smoke haze: three used an Indonesian Family Life survey, reporting the association of air pollution exposure from 1997 haze with lung capacity [53], cardiovascular disease prevalence [46], and cognitive function [45]; one examined lung function [100]; and the other used height as a nutritional outcome [55].

Health outcomes included all-cause mortality [52, 58, 63, 67, 82, 101, 103], respiratory diseases [63, 103], and cardiovascular diseases [103]. Jayachandran (2009) [52] examined the effects of smoke on infant mortality using an ecological design. Health outcomes other than mortality included clinic/hospital visits and hospitalization due to respiratory diseases [32, 54, 56, 57, 61, 62, 65, 66, 69, 76, 77, 84, 85, 98–100], cardiovascular diseases [61, 78, 80, 99], allergic diseases [32, 69, 76, 77], and lung cancer [64]. Several studies used information on symptoms obtained from interviews [50, 83], questionnaire surveys [45, 49, 51, 53, 54, 56, 75, 98], and reports from haze clinics [76]. Six studies examined lung function [53, 56, 70, 90, 98, 100], two examined cognitive function [45, 75], and two examined laboratory tests [53, 70].

The methods of exposure assessment varied according to the study. Binary variables indicating haze exposure are commonly used in analyses [51, 54, 58, 61, 63–67, 69, 70, 75, 98]. A haze episode was defined according to a certain cutoff value of PM10 [58, 63, 66, 69], visibility [58, 64, 65], or aerosol index derived from satellite data [51]. Two studies considered different aspects of exposure: duration and intensity [67], and days with burning activities [32]. Few studies used a binary variable specified by the time period [61, 83, 98]. Studies from Singapore used a categorical variable based on the PSI [38, 78–80]. Studies from Thailand have generally used PM10 as a continuous variable [32, 90, 97, 99, 101, 103]; whereas other studies used aerosol index values derived from satellite data [52, 53, 55].

#### 3.2.2 Health burden estimation approach (HBE)

**Fig 4:**
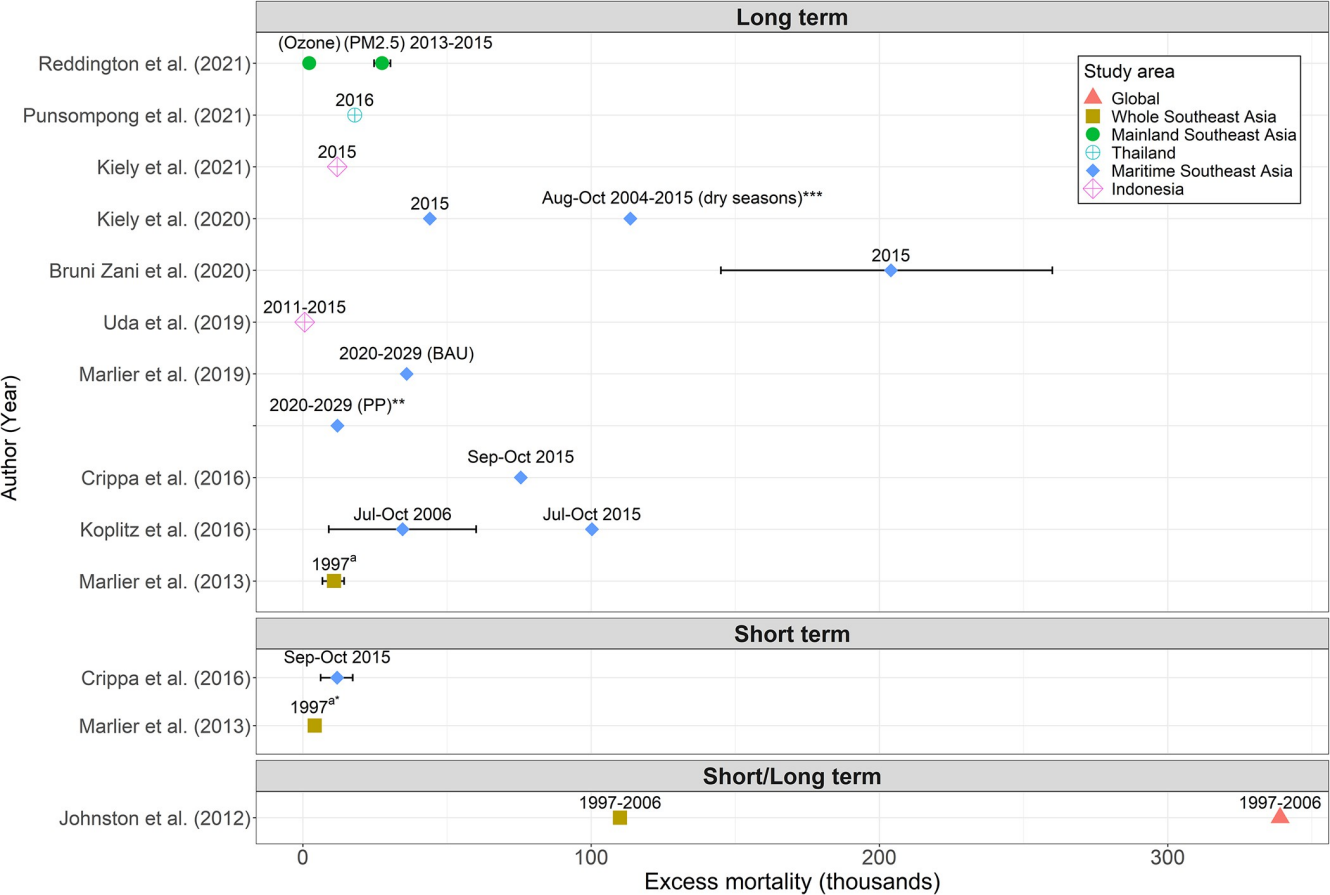
Excess mortality reported in health burden estimation studies in Southeast Asia using concentration-risk functions for long-term (top), short-term (middle), and both short- and long-term (bottom) exposures. ^a^ denotes cardiovascular mortality. * denotes the health burden of ozone exposure. ** denotes an averaged estimate value. *** denotes health burden estimates from August to October of 2004, 2006, 2009, 2012, 2014, and 2015. BAU: *business-as-usual* scenario. PP: *protecting peatland* scenario.

Eleven HBE studies estimated the health burden of vegetation and peatland fires in Southeast Asia. Among these, two studies included both global- and regional-scaled estimations, whereby Southeast Asia was one of the regions in the study [19, 105], six were regional-scaled (Southeast Asian region) [40, 86–89, 106], and three were conducted in a single country (Indonesia [41, 47] and Thailand [104]) (Fig 4). The burden estimation was based on historical estimation [19, 47, 86–89, 104–106] or future or scenario projections [40, 41] (S3 Table).

The health burden was reported as excess mortality for all-causes [19, 40, 41, 86–89, 105], chronic respiratory diseases [47, 86, 88, 104], lung cancer [47, 86, 88, 104], cardiovascular diseases [47, 86, 88, 104, 106], acute lower respiratory infection [40, 41, 89, 104, 105]. HBE studies included populations encompassing a wide age range; some included the whole population [19, 41, 47, 86, 88, 89, 104], while others targeted adults (age >25 years) [40, 47, 87, 105, 106] and under-fives [40, 47]. More than 300,000 deaths globally were estimated to be attributed to exposure to PM2.5 from vegetation fires during 1997–2006 [19], and approximately 100,000 deaths were reported in Southeast Asia during the fire seasons in 1997–2006 [19], 2004–2015 [89], and 2015 [87], with approximately 27,500 deaths in the mainland area [105]. Short-term exposure to fire-related ozone [106] and PM2.5 [86] was estimated to have resulted in 4,100 annual cardiovascular and 11,800 all-cause deaths, respectively, whereas long-term exposure to ozone has led to 2,250 excess deaths [105]. In the maritime area, long-term exposure to fire-related PM2.5 was expected to cause 100,300 [87], 75,600 [86], and 131,700 [89] all-cause deaths. Annual mortality in the maritime area also differed by year, depending on the occurrence and intensity of fires, and a study estimated 150,000 and 204,000 annual deaths in 2005 and 2015, respectively [88]. Meanwhile, the protection of fire-vulnerable areas can reduce preventable deaths. It was estimated that there would be fewer excess deaths (reducing 24,000 deaths) under the protecting peatland scenario (PP) compared to 36,000 excess deaths under the business-as-usual scenario (BAU) projected for 2020–2029 [40], and a 21% of excess deaths to be reduced under peatland restoration scenario [41].

The methods of exposure assessment in HBE studies included simulation of the pollutant of interest considering atmospheric conditions, such as fires or burning activities, and weather information. The emissions to be accounted for included all vegetation types [19, 40, 86–88, 104–106] or peatland [41, 47, 89]. Some studies specifically distinguished the haze period (e.g., July–August to September–October) and non-haze periods (e.g., November–December, January–July) to quantify the health burden distinct from different sources [86, 87, 89]; whereas, others simulated the annual average pollutant concentration that accounted for vegetation and peatland fires [19, 40, 41, 47,88, 104–106]. Sensitivity tests for exposure assessment included varying inputs of fire emissions [19] and meteorological conditions [87] for fire-related PM simulations.

All HBE studies applied concentration-response functions (CRFs) for PM2.5, except for two studies [105, 106] that applied CRFs for ozone based on previous epidemiological studies. These included four studies using long-term CRFs [40, 41, 47, 87–89, 104, 105], three studies using short- and long-term CRFs separately [86, 106], and one study that presented the combined excess mortality using both short- and long-term CRFs [19] (Fig 4). The counterfactual concentrations (i.e., concentrations beyond which there would be assumed the same risks as that of the minimum or maximum concentration) considered through CRFs were 5–200 μg/m^3^ for short-term [19] and <50 μg/m^3^ for long-term-PM2.5 exposures [19, 87], and a range of 6.96–8.38 μg/m^3^ depending on the specific disease [104]. For sensitivity tests, models were altered with different CRFs, such as by shifting between linear and log-linear functions [19, 47, 106].

#### 3.2.3 Combined epidemiology and health burden estimation (EPI+HBE) approach

Two studies used a combination of EPI and HBE approaches [107, 108] (S4 Table). Both studies estimated smoke-haze-attributable mortality globally; whereby, one study included Thailand and the Philippines [107], and another study included all countries in the Southeast Asia [108]. These studies first derived a CRF using an epidemiological approach, and subsequently used the CRF in the second part, the HBE approach, to estimate attributable mortality. Over short-term exposure to fire-related PM2.5, Chen et al. (2021) [107] estimated 33,510 all-cause, 6,993 cardiovascular, and 3,503 respiratory excess deaths globally. Another study [108] showed that long-term exposure to fire-related PM2.5 attributed to 12.9 million and 55,904 excess child mortality, globally and in Southeast Asia, respectively. Among Southeast Asian countries, Indonesia has comprised the highest number of excess child mortality [108].

#### 3.2.4 Health risk assessment approach (HRA)

Among the 15 HRA studies identified (S5 Table), nine were conducted in the maritime area (Malaysia [29, 59, 60, 68], Indonesia [48], and Singapore [71–74]), and six were conducted in Thailand [91–96] (i.e., mainland). Higher concentrations of PM10 [73, 91, 96], PM2.5 [29, 74, 95, 96], PAHs [73, 96], carcinogenic metals [48], and elemental potassium and secondary inorganic aerosols (i.e., indicators of biomass burning sources) [29] during haze compared to non-haze periods were reported. Peatland fires were linked to an extremely high level of PM2.5 (7,818 μg/m^3^ on October 1, 2009) in the immediate vicinity of the fire source (10–20 m) [48] compared with distant areas (54–329 μg/m^3^) [71].

Haze episodes pose potential carcinogenic [48, 71, 72, 74, 96] and non-carcinogenic risks [29, 48] to exposed populations. These risks have been demonstrated across different age groups [29, 60]. Carcinogenic risks increased with increasing intensity of haze [94], and these risks were observed for naturally ventilated indoor exposure, outdoor exposure, combined indoor and outdoor exposures [74], and in areas closer to burning activities [96]. One study highlighted the interactions between chemicals and lung fluids in the human body [72].

**Fig 5:**
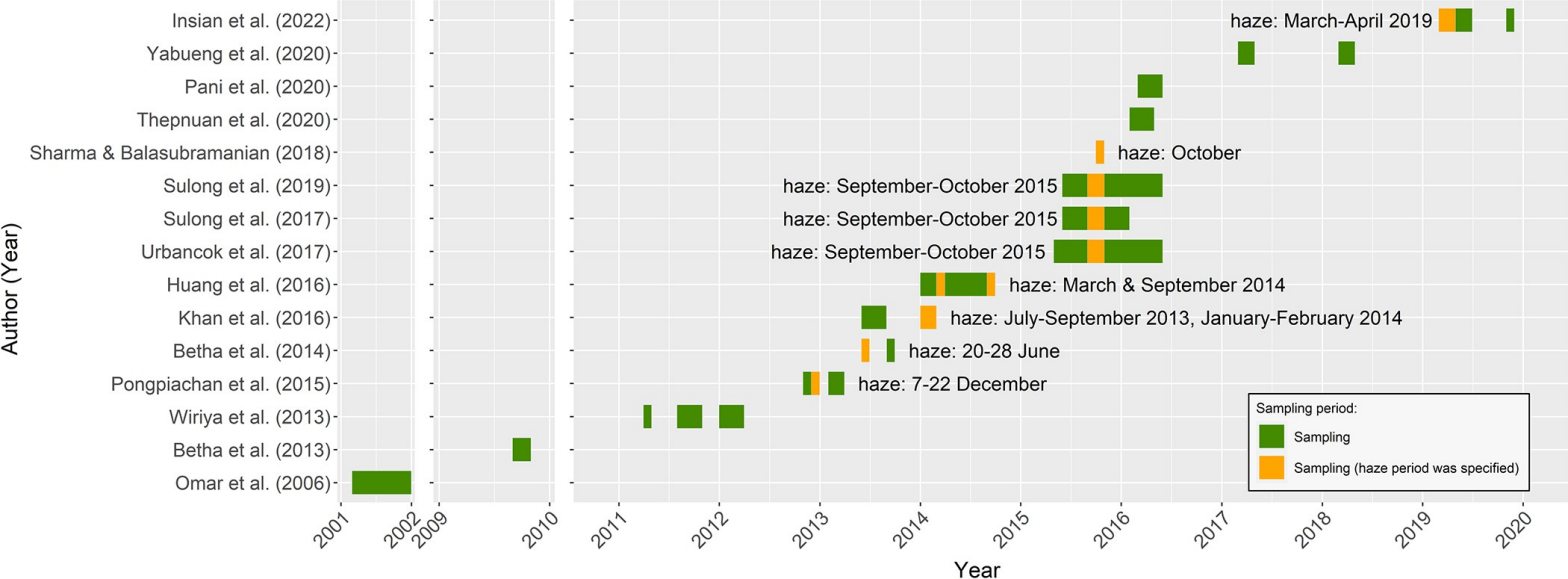
Timeline of sampling period and haze period in health risk assessment studies in Southeast Asia. Green indicates sampling period. Yellow indicates haze period specified within the sampling period.

Haze periods were determined for exposure assessment (Fig 5). Haze days were defined by PM2.5 concentration (PM2.5 >35 μg/m^3^) [29, 60], visibility (visibility <8, <6, and <3 km) [74], and air quality index (AQI >100) [73]. Some studies identified haze by areas [68, 95] or by burning activity seasons [59, 71, 72, 91–94, 96], during which samples were collected. Specifically, these studies identified haze events by examining burning sources [59, 95], weather conditions (i.e., dry or wet seasons) [91], and burning intensities (low: PM10 <50 μg/m^3^; medium: PM10 ranged 50–70 μg/m^3^; high: PM10 ranged 75–100μg/m^3^; and extreme: PM10 >100 μg/m^3^) [94]. One HRA study was conducted in the immediate vicinity of a fire source (peatland fire) [48].

Exposure-response assessments in HRA studies were classified into carcinogenic and non-carcinogenic. Cancer slope factor (SF) [48, 60, 72–74] or inhalation unit risk (IUR) [29, 59, 91, 92, 95, 96] was used for the carcinogenic assessment. Meanwhile, the reference dose (RfD) or reference concentration (RfC) was used [29, 48, 59, 72, 74] for the non-carcinogenic assessment. One study used the number of passively smoked cigarettes equivalent to a 1 μg/m^3^ increase in pollutants for both assessments [93].

Source apportionment was performed to identify the sources of chemicals or pollutants. There were five categories of chemical constituents: PAHs, trace metal elements, water-soluble ions, elemental (black) carbon, and biomass tracers (S5 Table). Biomass tracers, such as levoglucosan, mannosan, and galactosan, were used to determine whether vegetation or peatland fires contributed to the generation of chemicals rather than to characterize health risks. Source apportionment was based on fire hotspot data [59, 91, 93, 95], diagnostic ratio (DR) [60, 73, 91, 92, 94, 95], backward trajectory Hybrid Single-Particle Lagrangian Integrated Trajectory (HYSPLIT) model [71, 73, 91, 95], principal component analysis (PCA) [73, 91, 92], positive matrix factorization (PMF) [29, 59, 60], enrichment factor (EF) [59, 71, 72], aethalometer [93], BaA/CHR ratio [91], and BeP/(BeP+BaP) ratio [68].

### 3.3 Exposure levels and AQI

**Fig 6:**
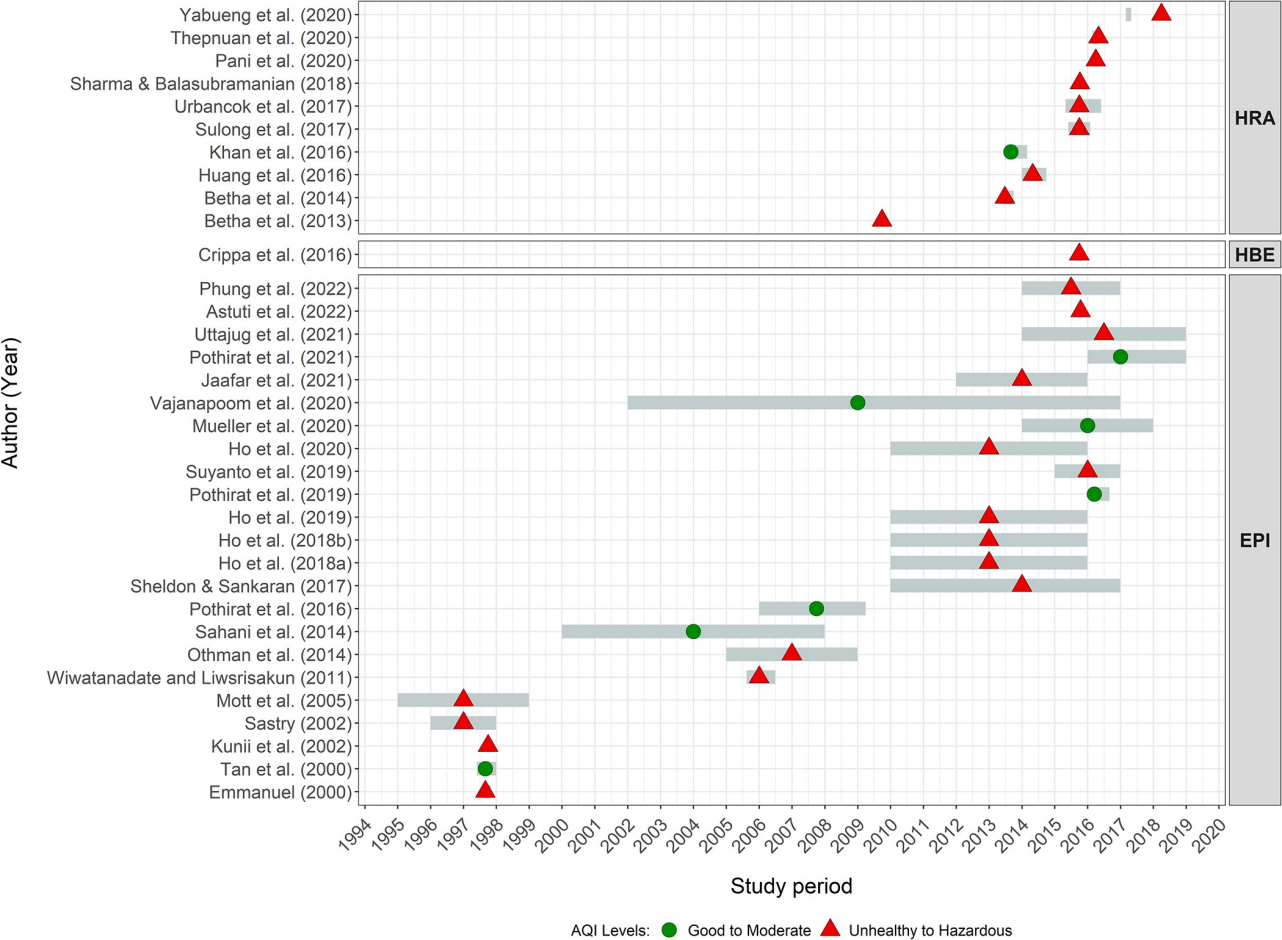
AQI levels by study period and study approach.

Most studies have reported exposure levels by different temporal dimensions (daily, monthly, seasonally, and annually) depending on the exposure assessment; the results are listed in Table 2. In some studies, such information could not be extracted because it was not available for several reasons: not reported due to study design (e.g., comparison of temporal trends); not reported for a specific study area, and thus the exposure quantification was directly made by spatial grids; or no exact value was available, and thus the results were displayed as figures. We identified studies for which the study period included the years 1997 [50, 58, 61, 69, 70], 2005 and 2006 [63, 90, 97, 101], 2009 [48], 2013 [59, 62, 71, 101], and 2015 [29, 32, 54, 57, 66, 67, 73, 74, 77–80, 82, 86, 99, 101], which were the years with severe regional smoke haze in Southeast Asia, especially in 1997, 2013, and 2015 (Fig 6).

We classified the AQI based on the U.S. EPA Air Quality Index [109] or the local AQI (e.g., PSI, API) if it was reported in the study. Consequently, AQI is indicated by the highest value among criteria pollutants observed on a daily scale; the studies that reported longer-term concentrations (e.g., monthly, seasonally, and annually) were not used for indications of AQI. We found 35 of the total 70 studies could be reanalyzed for AQI, whereby nine studies were classified as ‘good’ to ‘moderate’ AQI levels [59, 63, 70, 97–99, 101, 103] and 26 studies were classified as ‘unhealthy’ level, which encompasses the levels from ‘unhealthy for sensitive groups’ and above [29, 32, 48, 50, 54, 57, 58, 61, 62, 66, 67, 69, 71–74, 77–80, 82, 86, 90, 93–95] (Fig 6). Studies that were classified as ‘good’ to ‘moderate’ AQI levels might have had higher AQI levels within the study period, but this could not be identified in this study, and thus the reported values were based on a daily mean or median throughout the study period; the maximum value was not available. Most of the studies classified as ‘unhealthy’ AQI had reported the observed maximum level of pollution, while there were several studies which reported daily mean concentrations [29, 32, 62, 73, 74, 93–95] (Table 2). These high concentrations were mostly due to the haze period, as specified in the study by sampling period, or stratification by haze and non-haze periods.

## 4. Discussion

In this scoping review, we systematically identified studies on the health effects of smoke haze according to study approaches, such as EPI, HBE, and HRA. Although smoke haze is a regional issue in Southeast Asia, studies have been reported in majority of the countries in the region. All approaches revealed potential health risks due to smoke haze. Earlier works have mainly used descriptive designs in the EPI approach, especially after the severe Southeast Asia smoke haze episode in 1997. EPI studies in later years focused on estimating relative risks; however, most of these studies have a major limitation on exposure assessment. HBE studies have been conducted in recent years to quantify the smoke haze attributable health burden; however, most of these studies utilized CRFs from studies conducted in other regions or non-smoke-haze-related CRFs (i.e., using CRFs from total PM2.5). This may have led to uncertainties in the estimation. Finally, the HRA approach has contributed different information about the health risks of smoke haze. Unlike EPI and HBE, HRA studies have reported potential carcinogenic and non-carcinogenic risks owing to the toxicity of chemical constituents during smoke haze.

We clarified the methods and interpretations of the findings in each approach for studies conducted in Southeast Asia and found that more studies are needed to clarify the following aspects. First, there is a need for further long-term exposure studies. Currently, there are limited EPI studies that examine long-term exposure, and such information is needed as CRF in HBE studies. Second, further studies evaluating smoke haze and carcinogenic health risks are required. Most HRA studies have reported potential carcinogenic risks due to smoke haze, but these have been less investigated in EPI and HBE studies. Third, explore smoke haze effects on cause-specific health outcomes. Most EPI studies have shown consistent respiratory health effects due to smoke haze, but other health outcomes such as cognitive function, diabetes, and birth-related outcomes are scarce, although these health outcomes have been associated with exposures to PM and its constituents [114–118].

### 4.1 Terminology for vegetation and peatland fires

Various terminologies have been used to describe vegetation and peatland fires. The terms included ‘wildfire’ or ‘bushfire’ [36, 37, 52, 88, 100, 107, 119–128]; ‘agricultural burning,’ ‘open burning,’ or ‘biomass burning’ [5, 23, 59, 91–95, 99, 104]; ‘vegetation fire,’ ‘peat fire,’ ‘peatland fire,’ or ‘vegetation and peat fire’ [21, 26, 27, 32, 46–48, 86, 89]; ‘forest fire’ [44, 45, 51, 57, 58, 63, 95, 129]; ‘forest and vegetation fire’ [105]; ‘landscape fire’ [19, 106, 108, 130]; ‘Indonesian fire’ [41]; ‘smog’ [97, 98]; ‘haze’ or ‘Southeast Asian haze’ [28–30, 39, 44, 50, 54, 55, 60, 63–69, 74–76, 78–81, 83, 101, 131]; ‘smoke’ or ‘smoke haze’ [20, 40, 68, 71–73, 87, 94–96, 127, 132]; and ‘transboundary haze’ [29, 38, 60, 62, 74, 133]. In the present review, the terms ‘haze’ or ‘smoke haze’ were used to represent extreme air pollution episodes due to burning activities on vegetation and peatlands [134, 135]. Notably, haze generally refers to high pollutant concentrations, especially PM, and low visibility, and is widely used to describe extreme air pollution episodes not limited to vegetation and peatland fires, but also for other sources from urban, industrial, and desert dust [136–139]. Nonetheless, it is common to refer to smoke haze as a vegetation and peatland fire-related air pollution episode in Southeast Asia [133, 140–142]. This may be due to smoke blanketing and reduced visibility conditions caused by smoldering fires in peatlands, which are usually intensified in dry weather [143]. It may also have been used to describe extreme air pollution that was contributed by different burning sources, whereby it was difficult to describe using specific terminology of fires (e.g., forest fire and agricultural burning) [95].

### 4.2 Health effects and interpretation of findings

The reported health effects and their interpretations varied according to approach. EPI studies have reported measures of association, such as relative risk (RR), odds ratio (OR), and excess risk (percentage change). These measures show the direction and strength of an association and are used to evaluate causal inference and comparability with cross-disciplinary studies [144]. HBE studies reported on the health burden attributable to the pollutant of interest, usually with attributable excess mortality. Other measures of health burden, such as years of life lost (YLL) and disability-adjusted life years (DALY), have also been reported [41, 89]. Although mortality reflects the overall impact of the pollutant of interest [121], YLL and DALY could be used for quantification from the perspectives of valuation and economic cost, which would be more informative for policy decision making [145]. HRA studies have reported toxicity or carcinogenicity risks related to PM composition. While toxicity risks were reported as a ratio (HQ) showing the possibility of any non-cancer health effect; carcinogenicity risks were reported as a probability of cancer (e.g., 1 in 1,000,000 persons) if the population was to be exposed to the investigated chemicals for a lifetime [59].

### 4.3 Exposure assessment

Haze exposure was quantified using several methods. Many EPI studies conducted in the maritime area used binary variables (i.e., haze and non-haze) [51, 55, 58, 62–67, 69], while most studies in northern Thailand used continuous variables [32, 90, 97, 99, 101, 103]. HBE studies quantified exposure to pollutants of interest [19, 47, 87, 89], such as fire-related PM [107, 108], and estimated population-weighted exposure [19, 41, 105]. Long-term exposure was estimated using the annual average pollutant concentration, and short-term exposure was estimated using the daily average pollutant concentration during specified periods that spanned several months to years. HRA studies quantified lifetime exposure to fire-related PM constituents through calculations that considered exposure duration and individual characteristics [29, 71]. For example, 60 haze days per year were used as an assumption when considering the worst-case scenario. Individual characteristics included the inhalation rate, body weight, age, and expected life years.

The main exposure variables differed for each approach. As shown in Fig 3, EPI studies in Southeast Asia comprised not only continuous pollutant variables but also binary and categorical indicators to quantify the health effects of smoke haze. This review found that the connection between EPI and HBE was mainly comprised of PM2.5 as an exposure indicator. Although HRA studies have focused on PM, the analyses were mainly based on PM constituents, which suggested both potential carcinogenic and non-carcinogenic toxicity related to smoke haze pollutants. However, no EPI studies have examined health effects related to the PM constituents.

### 4.4 Exposure-response association and assessment

Exposure-response association is a function which indicates health effects given a particular level of exposure. CRF is established through EPI studies and is applied in HBE studies to estimate the attributable health burden. One major difference between EPI and HBE/HRA studies is that the EPI approach aims to examine associations and causal inferences, whereas the HBE and HRA approaches assume that exposure is causally related to health outcomes.

In the present review, most CRFs applied in HBE studies were based on epidemiological studies in urban settings in other regions [19, 40, 47, 86]. This may have increased uncertainties owing to differences in pollutant emissions and chemical compositions of fires in different regions [1, 146]. Although an increasing number of studies have attempted to estimate the health burden of fire-related PM [147], only two studies have been conducted to estimate the attributable mortality for global and included Southeast Asia, comprising the entire population in Thailand and the Philippines [107]; and children in Southeast Asia [108]. In addition, while the HBE approach may be used to estimate health burden based on exposure duration, most epidemiological studies in Southeast Asia have focused on short-term exposure. Similar to the HBE approach, the HRA approach applies risk functions to assess health risks due to the pollutant of interest. Risk functions in HRA studies are often derived from animal studies, given the difficulty in conducting human studies which consider a lifetime period.

### 4.5 Research gaps and future studies

The present literature review revealed research gaps and challenges related to the interconnectivity of the three approaches. First, there was heterogeneity in the exposure assessment methods, which limited the connectivity and generalizability of the evidence. The HBE studies used population-scale exposure levels, and no individual exposure levels, which may differ according to the pattern of daily activities, were considered. Behavior and mitigation measures, such as school closure and reduction of outdoor movements, implemented during haze episodes may also lead to misclassification of actual exposure and increased uncertainty. In contrast, HRA studies accounted for individual characteristics, such as age, body weight, inhalation rate, and years of exposure. Although the EPI approach is relatively advantageous in terms of demonstrating associations based on observed datasets, long-term studies require extensive effort. In this sense, the HBE and HRA approaches may complement EPI studies, but these approaches require careful consideration of the underlying assumptions.

Second, there is little evidence regarding the health effects of various pollutants or chemical components released into smoke plumes. PM was among the most intensively studied pollutants in EPI and HBE studies; whereas HRA studies mainly examined the toxicity of PM constituents emitted during smoke haze [33, 127]. Emissions of fire-related pollutants may vary depending on vegetation type and burning conditions [2, 148]. Furthermore, it is difficult to distinguish and quantify fire-related pollutants from peatland fires because of the nature of the shift between flaming and smoldering condition [146]. HRA studies have shown potential carcinogenic risks of smoke haze, but only one EPI study [64] and two HBE studies [47, 104] have examined lung cancer risk. Black carbon was mentioned in one HBE study [87], and only one HRA study examined its health risk [93]. Gaseous pollutants such as carbon monoxide (CO) have been shown to increase the prevalence of headaches in EPI studies [56], but health risks due to exposure to such pollutants are yet to be clarified, especially in the vicinity of burning sites where the concentration of CO is high [149–152].

Third, the local and transboundary sources of smoke haze from vegetation and peatland fires remain largely unaddressed. The wind direction and dry season caused an imbalance in the amount of pollutants in the fire pollutant source and receptor areas. Some areas may not have burning activity but are exposed to high concentrations of transboundary pollutants. Local sources of haze pollutants can be reduced or controlled via local mitigation policies, but transboundary sources of haze pollutants require efforts across borders. Additionally, pollutants in burning areas may differ from those found in distant locations. Burning conditions such as moisture content and weather may contribute to this [2, 153], for example, higher EC, K+, CL-, and PAHs at flammable and higher temperatures; levoglucosan and water-soluble organic carbon at low temperatures and in smoldering combustions [154]. Thus, evidence across multiple areas in the region is needed to facilitate policy decision making.

Future studies should consider the interconnectivity between different approaches. Pollutants and chemicals quantified in HRA sampling may be further utilized in EPI studies, although more effort may be required given the need for a larger dataset. Findings reported in EPI and HRA studies regarding chemical components may be considered in exposure assessments in HBE studies. Studies with a combination of approaches, such as the EPI- and HBE-combined approach [107, 108], would be useful because they maximize the strengths of one approach and complement the limitations of the other. For example, the combined approach demonstrates both EPI evidence and health burden, which would facilitate future policy decisions and risk communication. More EPI studies compiling different local characteristics with similar exposure metrics could facilitate the quantification of risks and establish exposure-response functions to be applied in HBE studies in a particular region.

## 5. Conclusion

This study reviewed previous studies on smoke haze-related health effects in Southeast Asia. The studies were reviewed and discussed based on EPI, HBE, and HRA approaches. This study found that although all the approaches indicated potential health risks due to smoke haze, currently available studies have limited interconnectivity among approaches. This is due to the heterogeneity in exposure assessments, the use of different pollutants or exposure metrics, and the unaddressed issue of smoke haze sources.

Future studies should consider integrating the findings from the three approaches through study designs with comparable exposure assessments and a combination of approaches. The sources of smoke haze should be clearly indicated, as this would facilitate policy decisions for efficient mitigation of smoke haze in the region.

## Supporting information

S1 Checklist PRISMA-ScR checklist. (PDF)

S1 Table Search terms used in each search engine. (DOCX)

S2 Table Summary of epidemiological studies on the health effects of smoke haze in Southeast Asia. (DOCX)

S3 Table Summary of health burden estimation studies on the health effects of smoke haze in Southeast Asia. (DOCX)

S4 Table Summary of studies using combined epidemiological and health burden estimation approaches on the health effects of smoke haze in Southeast Asia. (DOCX)

S5 Table Summary of health risk assessment studies on the health effects of smoke haze in Southeast Asia. (DOCX)

S1 Appendix List of articles subjected for review. (DOCX)

S1 Graphical abstract (TIF)
