# Correction: A scoping review on the health effects of smoke haze from vegetation and peatland fires in Southeast Asia: Issues with study approaches and interpretation

**DOI:** 10.1371/journal.pone.0315698

**Published:** 2024-12-10

**Authors:** The PLOS ONE Staff

## Notice of republication

This article was republished on 22 November 2024, to correct an error in the byline that was introduced during the typesetting process. The second author’s last name was spelled incorrectly. The correct name is: Athicha Uttajug. The correct citation is: Phung VLH, Uttajug A, Ueda K, Yulianti N, Latif MT, Naito D (2022) A scoping review on the health effects of smoke haze from vegetation and peatland fires in Southeast Asia: Issues with study approaches and interpretation. PLoS ONE 17(9): e0274433. https://doi.org/10.1371/journal.pone.0274433

The publisher apologizes for the errors. Please download this article again to view the correct version. The originally published, uncorrected article and the republished, corrected articles are provided here for reference.

## Supporting information

S1 File Originally published, uncorrected article. (PDF)

S2 File Republished, corrected article. (PDF)
