# Supplementary material for: A scoping review on the health effects of smoke haze from vegetation and peatland fires in Southeast Asia: Issues with study approaches and interpretation
Source: PLoS One. 2022 Sep 15;17(9):e0274433. doi: 10.1371/journal.pone.0274433 (PMC9477317; doi:10.1371/journal.pone.0274433)
Supplement: S1 Table — (DOCX) [file pone.0274433.s002.docx]

**S1 Table. Search terms used in each search engine**

| **Search engine** | **Search terms** |
| --- | --- |
| **Pubmed** | ("forest fire*" OR "peatland fire*" OR "wildfire" OR "Prescribed fire" OR "Vegetation fire" OR "landscape fire" OR "Vegetation fire" OR "Agricultural burning" OR "Transboundary haze" OR "Smoke haze" OR "Biomass burning" OR "Bushfire") AND  ("Health" OR "Mortality" OR "Morbidity" OR "Hospital admission" OR "Emergency visit" OR "Out-patient" OR "emergency ambulance dispatch*" OR "Health risk assessment" OR "symptom*" OR "respiratory" OR "cardiovascular" OR "Cancer" OR "clinic visit*"OR "Public health" OR "health risk*" OR "Health impact" OR "Mental health" OR "Psychological" OR "death*" OR "birth-related" OR "asthma") AND  ("southeast asia" OR "ASEAN" OR "asia" OR "malaysia" OR "thailand" OR "indonesia" OR "laos" OR "myanmar" OR "cambodia" OR "vietnam" OR "singapore" OR "brunei" OR "philippines") |
| **Scopus** | ("forest fire" OR "peat land fire" OR "wildfire*" OR prescribed fire*" OR vegetation fire*" OR "landscape fire*" OR "agricultural fire*" OR "trans-boundary haze" OR "smoke haze" OR "biomass burning" OR "bushfire") AND  ("health" OR "mortality" OR "morbidity" OR "hospital admission" OR "emergency visit" OR "out-patient" OR "emergency ambulance dispatch*" OR "health risk assessment" OR "symptom*" OR "respiratory" OR "cardiovascular" OR "cancer*" OR "clinic visit*" OR "public health" OR "health risk*" OR "health impact" OR "mental health" OR "psychology" OR "premature death" OR "death*" OR “birth-related” OR “asthma”) AND  ("southeast asia" OR "ASEAN" OR "asia" OR "malaysia" OR "thailand" OR indonesia" OR "laos" OR "myanmar" OR "cambodia" OR "vietnam" OR singapore" OR "brunei" OR "philippines") |
| **Web Of Science** | (forest fire* OR peat land fire* OR wildfire* OR prescribed fire* OR vegetation fire* OR landscape fire* OR agricultural fire* OR agricultural burning OR trans-boundary haze OR smoke haze OR biomass burning OR bushfire) AND  (health OR mortality OR morbidity OR hospital admission* OR emergency visit* OR out-patient OR emergency ambulance dispatch* OR health risk assessment OR symptom* OR respiratory OR cardiovascular OR cancer* OR clinic visit* OR public health OR health risk* OR health impact* OR mental health OR psychological OR death OR birth-related OR asthma) AND  (south*east asia OR ASEAN OR asia* OR malaysia OR thailand OR indonesia OR laos OR myanmar OR cambodia OR vietnam OR singapore OR brunei OR philippines) |
