# Supplementary material for: A scoping review on the health effects of smoke haze from vegetation and peatland fires in Southeast Asia: Issues with study approaches and interpretation
Source: PLoS One. 2022 Sep 15;17(9):e0274433. doi: 10.1371/journal.pone.0274433 (PMC9477317; doi:10.1371/journal.pone.0274433)
Supplement: S2 Table — (DOCX) [file pone.0274433.s003.docx]

**S2 Table. Summary of epidemiological studies on the health effects of smoke haze in Southeast Asia**

| **Author (Year)** | **Study area** | **Study period** | **Exposure time** | **Study design** | **Data source** | **Subjects and health outcome** | **Exposure** | **Haze definition** | **Results** |
| --- | --- | --- | --- | --- | --- | --- | --- | --- | --- |
| Brauer and Hisham-Hashim (1998)[1] | Malaysia and Singapore | Aug–Sep 1997 | Short-term | Descriptive | Ministry of Health for Malaysia and Singapore | Number of hospital visits from respiratory disease | NA | Haze episode (Aug–Sep 1997) | Increased clinic visits due to respiratory diseases |
| Aditama (2000)[2] | Indonesia | Sep 1997–Jun 1998 | Short-term | Descriptive | Survey by physicians and reports from province/district health ofﬁces and hospitals | Respiratory diseases, (prevalence of bronchitis and bronchial asthma), and symptoms (eye, respiratory, headache) | NA | Haze disaster (Sep–Nov 1997) | During Sep–Nov 1997, number of deaths, asthma, bronchitis, and acute respiratory infections (ARI) were described (ARI cases increased by 1.8–3.8 times) |
| Emmanuel (2000)[3] | Singapore | Aug–Nov 1997 | Short-term | Time series analysis | Haze surveillance under Ministry of Health | Polyclinics visits (conjunctivitis, acute URTI, allergic rhinitis, acute bronchitis, asthma, eczema) | Haze defined PSI/PM10 | PM10 >50 μg/m^3^ | An increase in PM10 from 50 to 150 μg/m^3^ was associated with　increases of URTI (12%), asthma (19%), and rhinitis (26%) |
| Tan et al. (2000)[4] | Singapore | Jun–Dec 1997 | Short-term | Comparison between haze and post-haze period | Questionnaire, blood sample, and lung function test | Lung function, blood counts for national service men who participated the study | Haze | Haze period (Sep–Oct 1997), post-haze period (Nov–Dec 1997) | PM10 and SO_2_ were significantly associated with elevated band neutrophil counts. There was no difference in FEV1 and FVC during and post-haze period. |
| Odihi (2001)[5] | (Muara and Temburong) Brunei Darussalam | Sep 1997–Jun 1998 (haze); Jan–Jun 1997, Sep 1998 (after haze) | Short-term | Descriptive | Household interview (randomly sampled survey and group discussion) | Symptoms about COPD and respiratory diseases, and health condition (not described) | NA | Haze period (Sep 1997 and Jan–Jun 1998) | Survey showed certain health conditions appeared during haze period. The methods were not clearly shown. There was no descriptive table. |
| Kunii et al. (2002)[6] | Jambi, Indonesia | Sep 29, 1997, and Oct 7, 1997. | Short-term | Descriptive | Interview at 6 sites (school, nursing home, government office, and village) | Symptoms for the respondents | NA | 1997 haze (not clearly defined) | Almost all among the respondents had respiratory symptoms. More than half complained about eye symptoms, and headache, and fatigue. |
| Sastry (2002)[7] | (Kuala Lumpur, Johor Bahru, Ipoh, Kuching, and Penang) Malaysia | 1996–1997 (Target is 1997 Southeast Asian forest fires) | Short-term | Time series analysis | Mortality data from vital statistics records. | Mortality (all-age, <1 year, 1–64 years, 65–74 years, >75 years) | Smoke haze as defined by PM10 or visibility | PM10 >210 μg/m^3^, or visibility <0.91 km | Mortality, especially for the elderly was higher during smoke haze in Kuala Lumpur and Kuching (56%). Increased infant mortality (69%) was also observed in Kuala Lumpur during the day with lower visibility. |
| Anaman et al. (2003)[8] | (Muara) Brunei Darussalam | Haze episode in Jan-Apr 1998 | Short-term | Time series analysis | Ministry of Development and Health, hospitals, health centers, and clinics | Daily visits to hospitals, health centers and clinics respiratory diseases and conjunctivitis | PSI | Haze (Jan–Apr 1998) | The total daily number or health outcomes were significantly related to PSI. |
| Frankenberg et al. (2005)[9] | Indonesia | 1997 | Short-term | Cross-sectional study | Indonesia Family Life Survey (IFLS) | Self-reported health indicator (Physical function, cough, and general health conditions) for adults (age >30 years) for 7,200 households | Haze defined by TOMS aerosol index | the TOMS aerosol index exceeded 1.5 | Exposure to haze is associated with reduced physical function and respiratory symptoms. |
| Mott et al. (2005)[10] | (Kuching) Malaysia | 1995–1998 | Short-term | Comparison of observed and forecasted during haze period | Hospital admission data obtained from Sarawak Health Department | cardiorespiratory (cardiovascular, respiratory) hospitalizations from all public hospital admissions | Fire period | Fire period (Aug–Oct 1997) and non-fire period (Aug–Oct 1995, 1996, and 1998) | A significant fire-related increases were observed in respiratory hospitalizations, particularly COPD and asthma |
| Jayachandran (2009)[11] | Indonesia | 1997 | Short-term | Ecological study | Census of Population for Indonesia | Logarithm of (number of births) as a proxy for the early-life mortality rate | Smoke defined by TOMS aerosol index | TOMS index. 0.038 (non-smoke month), 0.578 (smoke month) | Air pollution from smoke was associated with decrease in birth cohorts. |
| Wiwatanadate et al. (2011)[12] | Chiang Mai, Thailand | Aug 15, 2005–Jun 30, 2006 | Short-term | Panel study | Obtained from the study participants who were asthmatic patients at the department of Internal Medicine of Chiang Mai University | Asthmatic symptoms and peak expiratory flow rate for asthmatic patients (age >12 years) | PM2.5, PM10, O_3_, NO_2_, SO_2_ | NA | Peal expiratory flow rate was positively associated with NO2, SO2, and PM10, which are contradictory to most of the previous studies. There were no association between symptoms. |
| Ho, R.C. et al. (2014)[13] | Singapore | Jun 21–26, 2013 | Short-term | Cross-sectional study | Collected via online questionnaire | Physical symptoms and psychological stress during the haze crisis for the participants by online recruitment and snowball sampling | Haze crisis (Jun 2013) | Haze crisis (Jun 2013) | Increased physical symptoms during the haze. Psychological stress was associated with physical symptoms and perceived dangerous PSI values. |
| Othman et al. (2014)[14] | Selangor, Malaysia | 2005–2006 and 2008–2009 | Short-term | Regression | Database by the Ministry of Health Malaysia via the Health Informatics Center | Respiratory and cardiovascular hospital admissions | API, PM10 | Haze episode (API >76) | A smoke haze occurrence was associated with an increase in inpatient cases. |
| Sahani et al. (2014)[15] | Klang Valley region , Malaysia | 2000–2007 | Short-term | Case-crossover design | Mortality data from Department of Statistics, Malaysia | Non-accidental mortality (respiratory, natural) | Haze event defined by PM10 | PM10 >100 μg/m^3^ | Haze events was associated with higher natural mortality. Exposure to haze events showed immediate and delayed effects on mortality. |
| Yeo et al.(2014)[16] | Singapore | Jun 25, 2013–July 11, 2013 | Short-term | Descriptive | Data was obtained from Haze clinic and Haze consultation. | Medical conditions for those who visited the haze clinic. (respiratory, eye and skin-related) | NA | Haze period (Jun–Jul of 2013) | Majority of haze-related complaints were respiratory, eye and skin-related. |
| Pothirat et al. (2016)[17] | (Chiang Mai) Thailand | Jan–Mar in 2006–2009 | Short-term | Poisson regression | Data collected from Chiang Mai University and Chiang Mai Ram hospitals | Daily emergency visits due to acute exacerbations of COPD and asthma | PM10 (during dry season: Jan-Mar) | Seasonal smog (Jan–Mar) | PM10 was associated with exacerbations of asthma and COPD. |
| Hassan et al. (2017)[18] | (Kuala Lumpur) Malaysia | Jan 2010–Oct 2015 | Short-term | Comparison between haze/non-haze period | Chart reviews and the hospital’s electronic database at Universiti Kebangsaan Malaysia Medical Centre (UKMMC) | Lung cancer for those presented at UKMMC | Haze defined by visibility | Visibility <10 km | Increased presentation due to respiratory symptoms and diagnosis of lung cancer during haze. |
| Kim et al. (2017)[19] | Indonesia | 1993, 1997, 2000, and 2007 | Long-term | Cohort study | Indonesian Family Life Survey (IFLS) for 1993, 1997, 2000, and 2007 | Subjective (general health status (GHS) and activities of daily living (ADL)) and objective (lung capacity and hemoglobin), and lung capacity (age >9 years) | TOMS aerosol index | 1997 haze (Sep–Nov 1997) | TOMS aerosol index at 1997 was inversely associated with lung capacity and positively associated with GHS and ADL in 2007. |
| Sheldon et al. (2017)[20] | (Center of Singapore) Singapore | 2010–2016 | Short-term | Regression | Weekly polyclinic attendance data was obtained from Singapore's Ministry of Health | Polyclinic attendances (acute upper respiratory tract infections (ARTIs), acute conjunctivitis (AC), acute diarrhea, and chickenpox) | PSI | NA | Increased PSI was associated with weekly polyclinic attendances for ARTIs and AC. |
| Syam et al. (2017)[21] | Borneo and Sumatra, Indonesia | Oct 2015–Nov 2015 | Short-term | Descriptive | Data obtained from online questionnaire | Various symptoms, asthma exacerbation, worsening or exacerbation of previous chronic disease | NA | Not specified | Increased headache, cough, rhinorrhea, sore throat, dyspnea; higher risk if less quality protective measure is used |
| Ho, A.F.W. et al. (2018a)[22] | Singapore | 2010–2015 | Short-term | Case-crossover design | Pan-Asian Resuscitation Outcomes Study (PAROS) registry | Out of hospital cardiac arrest (OHCA) occurrence | Haze categorized by PSI | Moderate PSI (51–100) and unhealthy PSI (101–200). | Moderate and unhealthy PSI levels were associated with an increase in OHCA. |
| Ho, A.F.W. et al. (2018b)[23] | Singapore | 2010–2015 | Short-term | Case-crossover design | Singapore Stroke Registry | Ischemic stroke occurrence | Haze categorized by PSI | Moderate PSI (51-100) and unhealthy PSI (101-200). | Moderate and unhealthy PSI levels were associated with an increase in stroke occurrence |
| Ming et al. (2018)[24] | (Klang Valley) Malaysia | 2014–2015 | Short-term | Comparison between haze/non-haze period | Record reviews and discharge registers of Universiti Kebangsaan Malaysia Medical Centre (UKMMC) | Respiratory admissions to UKMMC | Haze defined by visibility | Visibility <10 km | Higher respiratory admissions, possibility of ICU and longer stay for those who admitted during haze period than non-haze period. |
| Ho, A.F.W. et al. (2019)[25] | Singapore | 2010–2015 | Short-term | Case-crossover design | The Singapore Myocardial Infarction Registry | Acute myocardial infarction (AMI) occurrence | Haze categorized by PSI | Moderate PSI (51–100) and unhealthy PSI (101–200). | Moderate and unhealthy PSI levels were associated with an increase in AMI |
| Pothirat et al. (2019)[26] | (Chiang Dao district) Thailand | Mar and Aug 2016 | Short-term | Comparison between August and March | Survey of the questionnaire and lung function for the participants at Chiang Dao Hospital | QOL, Symptoms, lung function for COPD patients aged >40 years | Seasonal smog | Seasonal smog (March) | QOL and lung function (FVC and FEV1) were lower in March than August. |
| Suyanto et al. (2019)[27] | (Pekanbaru) Indonesia | 2015 (haze year); 2016 (post-haze year) | Short-term | Comparison between haze year/non-haze year, | Questionnaire survey for the study participants with newly confirmed tuberculosis aged 18-70 years old. | Perceived health condition measured by St George Respiratory Questionnaire | Haze year | Haze year (2015) | The subsequent respiratory morbidity status of tuberculosis (TB) cases was poorer among respondents treated during a haze year (2015) |
| Tan-Soo et al. (2019)[28] | (Sulawesi, Nusa Tenggara, Kalimantan, Sumatra) Indonesia | 1997, 2000, 2007, 2014; Exposure in Aug–Oct 1997 | Long-term | Cohort study | Indonesian Family Life Survey (IFLS) for 1997, 2000, 2007, and 2014 | Children average height-for-age z score (HAZ), as a nutritional outcome | TOMS aerosol index | Haze period (Aug–Oct 1997) | Higher aerosol index during 1997 haze was associated with decrease in HAZ at age of 17. |
| Aik et al. (2020)[29] | Singapore | 2009–2018 | Short-term | Time-series analysis | Weekly Infectious Disease Bulletin published by the Ministry of Health | Acute conjunctivitis | PM2.5, PM10 | Haze episodes (not described) | PM2.5 and PM10 were associated with increased risk of acute conjunctivitis. Haze episodes did not modify the relationship between PM2.5 or PM10 and acute conjunctivitis. |
| Ho, A.F.W. et al. (2020)[30] | Singapore | 2010-2015 | Short-term | Case-crossover design | Ministry of Home Aﬀairs’ Registry of Birth and Death | All-cause mortality | Haze categorized by PSI | Moderate PSI (51–100) and unhealthy PSI (101–200) | Moderate and unhealthy PSI levels were associated with an increase in mortality. |
| Mueller et al. (2020)[31] | (Upper north region) Thailand | 2014–2017 | Short-term | Time series analysis | Thailand Ministry of Public Health | Hospital visits due to respiratory and cardiovascular diseases (chronic lower respiratory disease, ischemic heart disease, cerebrovascular disease) | PM10 | Burning (Jan–Apr) and non-burning (May–Dec) period | Increased hospital visits for chronic lower respiratory disease and cerebrovascular disease, but not ischemic heart disease, were associated with higher PM10. |
| Ontawong et al. (2020)[32] | (Pong District, Phayao Province) Thailand | 4 years (No information on the study period) | Long-term | Panel study | Questionnaire and lung function survey conducted during the study period | Borg scale (intensity or severity of breathlessness) and lung function | Wildfire smoke | Not specified | FEV1 and FVC for the participants were lower than the normal range. |
| Vajanapoom et al. (2020)[33] | (Chiang Mai) Thailand | 2002–2016 | Short-term | Case-crossover design | Mortality data from the Ministry of Public Health. | Mortality (all-age, <1 year, 1–64 years, 65–74 years, >75 years) | PM10, haze control measures | NA | Examined the impacts of governmental haze control measures. Decreased PM10 concentration and percent excess risk per IQR PM10 after haze control measures. |
| Zaini et al. (2020)[34] | (Riau, Pekanbaru) Indonesia | 2015 | Short-term | Descriptive | Questionnaire survey conducted at 2 primary health centers | Symptoms and lung function for the respondents. | NA | Forest fire disaster in Oct 2015 | Not only respiratory but also ocular, gastrointestinal symptoms and headache were prevalent. Many had lung function impaired. |
| Jaafar et al (2021)[35] | (Selangor) Malaysia | 2012–2015 | Short-term | Cross-sectional study | Ministry of Health; and Universiti Malaya Medical Center | Admissions and visits to public healthcare facilities for patients diagnosed with acute exacerbation of bronchial asthma and chronic obstructive pulmonary disease | Haze defined by PM10 | PM10 >51 μg/m^3^ | There were more outpatient and inpatient patients due to respiratory diseases during the haze days compared to non-haze days. |
| Mueller et al. (2021)[36] | Thailand | Jan 1, 2015–Apr 30, 2018 | Long-term (during pregnancy) | Semi-ecological study design | Ministry of Public Health | Birth weight | PM10 | Sum of the fire spots detected by satellite data | Exposure to biomass burning during pregnancy was associated with reduced birth weight. |
| Pothirat et al. (2021)[37] | (Chiang Mai) Thailand | 2016–2018 | Short-term | Poisson regression | Data extracted from the bureau of Registration Administration of Chiang Mai | Non-accidental and cause-specific mortality | PM2.5, PM10 | NA | PM2.5 and PM10 were associated with mortality from non-accidental, COPD, coronary artery disease and sepsis. |
| Uttajug et al. (2021)[38] | (8 provinces in upper north region) Thailand | 2014–2018 | Short-term | Case-crossover design | Hospital visits data obtained from the Ministry of Public Health (MOPH), Thailand. | Hospital visits due to respiratory diseases, conjunctivitis, and dermatitis for children (1 month < age <15 years) | PM10 | Burning day (number of ﬁre hotspots > 90^th^ percentile and PM10 > 100 μg/m^3^ | PM10 concentration on a burning day was associated with an increase in respiratory disease-related hospital visit. |
| Astuti et al. (2022)[39] | (Palangka Raya, Central Kalimantan)  Indonesia | Oct 2015 | Short-term | Descriptive | Primary Care under Public Health Office of Palangka Raya City | Children with acute respiratory infection (ARI) | NA | Haze episode | The distribution pattern of children with ARI in Central Kalimantan during the forest fire in October 2015 was in cluster form. |
| Jalaludin et al. (2022)[40] | Indonesia | 2000, 2007/2008, and 2014/2015 | Long-term | Cohort study | Indonesia Family Life Survey (IFLS) for 2000, 2007/2008, and 2014/2015 | Cognitive function measured by Ravens Colored Progressive Matrices (RCPM) for children with the ages of 7–24 years | PM2.5 | NA | Although there were no difference in RCPM scores for children living in forest fire-prone provinces compared with children living in non-forest fire-prone provinces, RCPM scores were lower for children who had lived in a forest fire-prone province all life compared with children who lived in a non-forest fire-prone province all life. |
| Phung et al. (2022)[41] | (12 districts) Malaysia | 2014–2016 | Short-term | Case-crossover design | Family Health Development Division, Ministry of Health | Under-5 mortality | PM10 | PM10 >50 μg/m^3^; several intensity and duration | Null association between haze and under-5 mortality. |
| Siregar et al. (2022)[42] | (Sumatra Island) Indonesia | 2000–2007 (IFLS4: 2007/2008) | Long-term | Cross-sectional study | Indonesia Family Life Survey (IFLS) | Cardiovascular disease prevalence for 2324 residents of Sumatra who completed the chronic condition questionnaire | PM2.5 | NA | Annual average PM2.5 level was associated with 29% higher odds of having CVD prevalence. |

ARI: acute respiratory infection; URTI: upper respiratory tract infection; FEV1: forced expiratory volume in 1 s; FVC: forced vital capacity; TOMS: total ozone mapping spectrometer; COPD: chronic obstructive pulmonary disorder; PSI: pollutant standard index; API: air pollutant index; ICU: intensive care unit; QOL: quality of life; IQR: inter-quartile range; PM2.5, and PM10: particulate matter with aerodynamic diameter below 2.5μm and 10μm, respectively; SO_2_: sulfur dioxide; O_3_: ozone; NO_2_: nitrogen dioxide; CVD: cardiovascular disease.

References:

1. Brauer M, Hisham-Hashim J. Fires in Indonesia: Crisis and reaction. Environ Sci Technol. 1998;32: 13–16. doi:10.1021/es983677j

2. Aditama TY. Impact of haze from forest fire to respiratory health: Indonesian experience. Respirology. 2000;5: 169–174. doi:10.1046/j.1440-1843.2000.00246.x

3. Emmanuel SC. Impact to lung health of haze from forest fires: The Singapore experience. Respirology. 2000;5: 175–182. doi:10.1046/j.1440-1843.2000.00247.x

4. Tan WC, Qiu DW, Liam BL, Ng TP, Lee SH, van Eeden SF, et al. The human bone marrow response to acute air pollution caused by forest fires. Am J Respir Crit Care Med. 2000;161: 1213–1217. doi:10.1164/ajrccm.161.4.9904084

5. Odihi JO. Haze and Health in Brunei Darussalam: The Case of the 1997‐98 Episodes. Singap J Trop Geogr. 2001;22: 38–51. doi:10.1111/1467-9493.00092

6. Kunii O, Kanagawa S, Ismail ITS, Kunii O, Yajima I, Hisamatsu Y, et al. The 1997 haze disaster in indonesia: Its air quality and health effects. Arch Environ Health. 2002;57: 16–22. doi:10.1080/00039890209602912

7. Sastry N. Forest fires, air pollution, and mortality in Southeast Asia. Demography. 2002. pp. 1–23. doi:10.2307/3088361

8. Anaman KA, Ibrahim N. Statistical estimation of dose-response functions of respiratory diseases and societal costs of haze-related air pollution in Brunei Darussalam. Pure Appl Geophys. 2003;160: 279–293. doi:10.1007/s00024-003-8778-3

9. Frankenberg E, McKee D, Thomas D. Health consequences of forest fires in Indonesia. Demography. 2005;42: 109–129. doi:10.1353/dem.2005.0004

10. Mott JA, Mannino DM, Alverson CJ, Kiyu A, Hashim J, Lee T, et al. Cardiorespiratory hospitalizations associated with smoke exposure during the 1997 Southeast Asian forest fires. Int J Hyg Environ Health. 2005;208: 75–85. doi:10.1016/j.ijheh.2005.01.018

11. Jayachandran S. Air quality and early-life mortality: Evidence from Indonesia’s wildfires. J Hum Resour. 2009;44: 916–954. doi:10.1353/jhr.2009.0001

12. Wiwatanadate P, Liwsrisakun C. Acute effects of air pollution on peak expiratory flow rates and symptoms among asthmatic patients in Chiang Mai, Thailand. Int J Hyg Environ Health. 2011;214: 251–257. doi:10.1016/j.ijheh.2011.03.003

13. Ho RC, Zhang MW, Ho CS, Pan F, Lu Y, Sharma VK. Impact of 2013 South Asian haze crisis: Study of physical and psychological symptoms and perceived dangerousness of pollution level. BMC Psychiatry. 2014;14: 81. doi:10.1186/1471-244X-14-81

14. Othman J, Sahani M, Mahmud M, Sheikh Ahmad MK. Transboundary smoke haze pollution in Malaysia: Inpatient health impacts and economic valuation. Environ Pollut. 2014;189: 194–201. doi:10.1016/j.envpol.2014.03.010

15. Sahani M, Zainon NA, Wan Mahiyuddin WR, Latif MT, Hod R, Khan MF, et al. A case-crossover analysis of forest fire haze events and mortality in Malaysia. Atmos Environ. 2014;96: 257–265. doi:10.1016/j.atmosenv.2014.07.043

16. Yeo B, Liew CF, Oon HH. Clinical experience and impact of a community-led volunteer atmospheric haze clinic in Singapore. Southeast Asian J Trop Med Public Health. 2014;45: 1448–53. Available: http://www.ncbi.nlm.nih.gov/pubmed/26466431

17. Pothirat C, Tosukhowong A, Chaiwong W, Liwsrisakun C, Inchai J. Effects of seasonal smog on asthma and COPD exacerbations requiring emergency visits in Chiang Mai, Thailand. Asian Pacific J Allergy Immunol. 2016;34: 284–289. doi:10.12932/AP0668

18. Hassan A, Latif MT, Soo CI, Faisal AH, Roslina AM, Andrea YLB, et al. Short communication: Diagnosis of lung cancer increases during the annual southeast Asian haze periods. Lung Cancer. 2017;113: 1–3.

19. Kim Y, Knowles S, Manley J, Radoias V. Long-run health consequences of air pollution: Evidence from Indonesia’s forest fires of 1997. Econ Hum Biol. 2017;26: 186–198. doi:10.1016/j.ehb.2017.03.006

20. Sheldon TL, Sankaran C. The impact of Indonesian forest fires on Singaporean pollution and health. Am Econ Rev. 2017;107: 526–529. doi:10.1257/aer.p20171134

21. Syam AF, Elina A, Hapsari FCP, Rahardja C, Makmun D. Relation Between Exposure of Rainforest Fire Smoke and Clinical Complaints During Indonesia Rainforest Fire in September–October 2015. Adv Sci Lett. 2017;23: 6739–6742. doi:10.1166/asl.2017.9385

22. Ho AFW, Wah W, Earnest A, Ng YY, Xie Z, Shahidah N, et al. Health impacts of the Southeast Asian haze problem – A time-stratified case crossover study of the relationship between ambient air pollution and sudden cardiac deaths in Singapore. Int J Cardiol. 2018;271: 352–358. doi:10.1016/j.ijcard.2018.04.070

23. Ho AFW, Zheng H, De Silva DA, Wah W, Earnest A, Pang YH, et al. The relationship between ambient air pollution and acute ischemic stroke: A time-stratified case-crossover study in a city-state with seasonal exposure to the Southeast Asian haze problem. Ann Emerg Med. 2018;72: 591–601. doi:10.1016/j.annemergmed.2018.06.037

24. Ming CR, Ban Yu-Lin A, Abdul Hamid MF, Latif MT, Mohammad N, Hassan T. Annual Southeast Asia haze increases respiratory admissions: A 2-year large single institution experience. Respirology. 2018;23: 914–920. doi:10.1111/resp.13325

25. Ho AFW, Zheng H, Earnest A, Cheong KH, Pek PP, Seok JY, et al. Time-stratified case crossover study of the association of outdoor ambient air pollution with the risk of acute myocardial infarction in the context of seasonal exposure to the Southeast Asian haze problem. J Am Heart Assoc. 2019;8: e011272. doi:10.1161/JAHA.118.011272

26. Pothirat C, Chaiwong W, Liwsrisakun C, Bumroongkit C, Deesomchok A, Theerakittikul T, et al. Influence of particulate matter during seasonal smog on quality of life and lung function in patients with chronic obstructive pulmonary disease. Int J Environ Res Public Health. 2019;16: 106. doi:10.3390/ijerph16010106

27. Suyanto S, Geater A, Chongsuvivatwong V. The effect of treatment during a haze/post-haze year on subsequent respiratory morbidity status among successful treatment tuberculosis cases. Int J Environ Res Public Health. 2019;16: 4669. doi:10.3390/ijerph16234669

28. Tan-Soo JS, Pattanayak SK. Seeking natural capital projects: Forest fires, haze, and early-life exposure in Indonesia. Proc Natl Acad Sci U S A. 2019;116: 5239–5245. doi:10.1073/pnas.1802876116

29. Aik J, Chua R, Jamali N, Chee E. The burden of acute conjunctivitis attributable to ambient particulate matter pollution in Singapore and its exacerbation during South-East Asian haze episodes. Sci Total Environ. 2020;740: 140129. doi:10.1016/j.scitotenv.2020.140129

30. Ho AFW, Zheng H, Cheong KH, En WL. The relationship between air pollution and all-cause mortality in Singapore. Atmosphere (Basel). 2020;11: 9.

31. Mueller W, Loh M, Vardoulakis S, Johnston HJ, Steinle S, Precha N, et al. Ambient particulate matter and biomass burning: an ecological time series study of respiratory and cardiovascular hospital visits in northern Thailand. Environ Heal. 2020;19: 77. doi:10.1186/s12940-020-00629-3

32. Ontawong A, Saokaew S, Jamroendararasame B, Duangjai A. Impact of long-term exposure wildfire smog on respiratory health outcomes. Expert Rev Respir Med. 2020;14: 527–531. doi:10.1080/17476348.2020.1740089

33. Vajanapoom N, Kooncumchoo P, Thach TQ. Acute effects of air pollution on all-cause mortality: A natural experiment from haze control measures in Chiang Mai Province, Thailand. PeerJ. 2020;2020: 1–15. doi:10.7717/peerj.9207

34. Zaini J, Susanto AD, Samoedro E, Bionika VC, Antariksa B. Health consequences of thick forest fire smoke to healthy residents in Riau, Indonesia: A cross-sectional study. Med J Indones. 2020;29: 58–63. doi:10.13181/mji.oa.204321

35. Jaafar H, Azzeri A, Isahak M, Dahlui M. The impact of haze on healthcare utilizations for acute respiratory diseases: Evidence from Malaysia. Front Ecol Evol. 2021;9: 764300. doi:10.3389/fevo.2021.764300

36. Mueller W, Tantrakarnapa K, Johnston HJ, Loh M, Steinle S, Vardoulakis S, et al. Exposure to ambient particulate matter and biomass burning during pregnancy: associations with birth weight in Thailand. J Expo Sci Environ Epidemiol. 2021;31: 672–682. doi:10.1038/s41370-021-00295-8

37. Pothirat C, Chaiwong W, Liwsrisakun C, Bumroongkit C, Deesomchok A, Theerakittikul T, et al. The short-term associations of particular matters on non-accidental mortality and causes of death in Chiang Mai, Thailand: a time series analysis study between 2016-2018. Int J Environ Health Res. 2021;31: 538–547. doi:10.1080/09603123.2019.1673883

38. Uttajug A, Ueda K, Oyoshi K, Honda A, Takano H. Association between PM10 from vegetation fire events and hospital visits by children in upper northern Thailand. Sci Total Environ. 2021;764: 142923. doi:10.1016/j.scitotenv.2020.142923

39. Astuti Y, Permana I, Bayu R, Rahmawati H. Distribution pattern of children with acute respiratory infection during forest fire at Central Kalimantan Indonesia. Bangladesh J Med Sci. 2022;21: 171–174. doi:10.3329/bjms.v21i1.56345

40. Jalaludin B, Garden FL, Chrzanowska A, Haryanto B, Cowie CT, Lestari F, et al. Associations between ambient particulate air pollution and cognitive function in Indonesian children living in forest fire–prone provinces. Asia Pacific J Public Heal. 2022;34: 96–105. doi:10.1177/10105395211031735

41. Phung VLH, Ueda K, Sahani M, Seposo XT, Wan Mahiyuddin WR, Honda A, et al. Investigation of association between smoke haze and under-five mortality in Malaysia, accounting for time lag, duration and intensity. Int J Epidemiol. 2022;51: 155–165. doi:10.1093/ije/dyab100

42. Siregar S, Idiawati N, Pan WC, Yu KP. Association between satellite-based estimates of long-term PM2.5 exposure and cardiovascular disease: evidence from the Indonesian Family Life Survey. Environ Sci Pollut Res. 2022;29: 21156–21165. doi:10.1007/s11356-021-17318-4
