# Supplementary material for: A scoping review on the health effects of smoke haze from vegetation and peatland fires in Southeast Asia: Issues with study approaches and interpretation
Source: PLoS One. 2022 Sep 15;17(9):e0274433. doi: 10.1371/journal.pone.0274433 (PMC9477317; doi:10.1371/journal.pone.0274433)
Supplement: S3 Table — (DOCX) [file pone.0274433.s004.docx]

**S3 Table. Summary of health burden estimation studies on the health effects of smoke haze in Southeast Asia**

| **Author (Year)** | **Study area** | **Study period** | **Pollutant** | **Exposure assessment** | **Exposure time (short/long)** | **Exposure level** | **Population** | **Concentration-response function** | **Health outcome** | **Burden estimation** | **Sensitivity analysis** |
| --- | --- | --- | --- | --- | --- | --- | --- | --- | --- | --- | --- |
| Johnston et al. (2012)[1] | Global and sub-regions | 1997–2006 | PM2.5 | GEOS-Chem global 3-D chemical transport model, and AOD (MODIS, MISR) | Long-term exposure if an area (exposure cell) is affected by smoke for >3 months in >5 of the study years;  Short-term exposure for other areas | Short-term: PM2.5 ranged 5-200 μg/m^3^ (PM2.5 in grid cells with PM2.5 <5 μg/m^3^ were excluded; PM2.5 in grid cells with PM2.5 >200 μg/m^3^ were fixed as 200 μg/m^3^);  Long-term: PM2.5 <50 μg/m^3^ (PM2.5 in grid cells with PM2.5 >50 μg/m^3^ were fixed as 50 μg/m^3^) | Global whole populations; Sub-region populations | Short-term:  Linear,  $0.11\%$  $(95\% CI:0, 0.26\%)$; per 1 μg/m^3^ increment for PM2.5 concentration range 5-200 μg/m^3^  (Morgan et al. 2010; Sastry 2002; Hanninen et al. 2009)[2–4]  Long-term:  Linear,  $0.64\% (95\% CI:$  $0.35, 0.94\%)$;  per 1 μg/m^3^ increment for PM2.5 concentration; maximum effect fixed at PM2.5 concentration of 50 μg/m^3^  (Pope et al. 1995)[5] | Mortality (all-cause) | Global mortality = 339,000;  Sub-Saharan Africa = 157,000; Southeast Asia = 110,000 | Varying:   - Estimated PM2.5 concentrations - Pattern of exposure - Shape of CRF - Maximum yearly average exposure to estimate chronic exposure burden - Range of minimum and maximum daily exposures used to estimate sporadic exposure burden |
| Marlier et al. (2013)[6] | Southeast Asia | 1997–2006 | PM2.5; ozone | NASA GISS-E2 PUCCINI GCM and GEOS-Chem CTM models | Short-term: 24-h PM2.5; Ozone concentration at 1-2 p.m. as proxy for 8-h max and 24-h concentration;  Long-term: Annual average of PM2.5 |  | Adult (age >30 years) | Power, ${RR}_{(PM2.5)}=1+\alpha\left( I\times C \right)^{\beta};$  $\alpha$ = 0.2685; $\beta$ = 0.2730 (Pope et al. 2011)[7]  Linear,  ${RR}_{(ozone)}=exp\left[ \delta\left( C_{fire}-C_{nofire} \right) \right]$  $\delta$ = 1.11(0.68-1.53); per 10 ppb increment in 24-h ozone concentrations (Bell et al. 2005)[8] | Cardiovascular mortality | In high fire year (1997):  PM2.5 attributable annual mortality = 10,800;  Ozone attributable annual mortality = 4,100 | Log-linear,  Cardiopulmonary  ${RR}_{(PM2.5)}=\left[ \frac{\left( C_{fire}+1 \right)}{\left( C_{nofire}+1 \right)} \right]^{\gamma}$  (Ostro 2004)[9];  $\gamma$ = 0.1551(0.05624-0.2541); C is annual average PM2.5 concentration  Linear,  ${RR}_{(PM2.5)}=exp\left[ \delta\left( C_{fire}-C_{nofire} \right) \right]$;  $\delta$ = 0.0128(0.0077-0.0182); concentration range 5.8-30 μg/m^3^  (Krewski et al. 2009)[10]  Short-term exposure to PM10 (per 10 μg/m^3^ increment) (PM10 derived from PM2.5 with a ratio of 0.6),  Linear,  ${RR}_{(PM2.5)}=exp\left[ \delta\left( C_{fire}-C_{nofire} \right) \right]$;  $\delta$ = 1% (0.6-1.5%); concentration upper threshold 125 μg/m^3^ (Ostro 2004)[9] |
| Crippa et al. (2016)[11] | Maritime Southeast Asia (Sumatra, Borneo, and Singapore) | 2015 | PM2.5 | WRF-Chem | Short-term: 24-h PM2.5 (Sep-Oct 2015);  Long-term: Annual average for fire (Sep–Oct) and non-fire (other months) periods | Areas with PSI > 101 (“unhealthy”, “very unhealthy”, “hazardous” levels) for >50% of Sep-Oct 2015 period | Whole populations;  Age-and cause-specific for long-term exposure estimation | Short-term:  $RR=exp\left[ \beta\times\left( {PM}_{fire}-{PM}_{nofire} \right) \right]$;  $\beta$ = 0.00104; per unit increment in PM2.5 (Atkinson et al. 2014)[12]  Long-term: Integrated exposure-response function (IER) (Burnett et al. 2014)[13] | Mortality (all-cause for short-term; cause specific for long-term (COPD, IHD, LC, and Stroke)) | Mortality from short-term exposure = 11,880;  Mortality from long-term exposure = 75,589 | NA |
| Koplitz et al. (2016)[14] | Maritime Southeast Asia (Indonesia, Malaysia, and Singapore) | Jul–Oct in 2006 and 2015 | PM2.5 | GEOS-Chem global 3-D CTS model; Population-weighted PM2.5 concentration (annual exposure derived from the averaged PM2.5 in Jul–Oct) | Long term: Annual average of PM2.5 | <50 μg/m^3^ | Adults (age >25 years) | Long-term: 1% increase in annual all-cause mortality per 1 μg/m^3^ increment in the annual average PM2.5 concentrations, up to 50 μg/m^3^ (Schwartz et al. 2008; Anenberg et al. 2012; Lepeule et al. 2012)[15–17] | Mortality (all-cause) | Mortality in 2006 = 37,600 (34,600 in Indonesia; 2,300 in Malaysia; 700 in Singapore);  Mortality in 2015 = 100,300 (91,600 in Indonesia; 6,500 in Malaysia; 2,200 in Singapore)  Regional smoke-related mortality in 2015 was 2.7 times higher than in 2006 | NA |
| Marlier et al. (2019)[18] | Maritime Southeast Asia (Indonesia, Malaysia, and Singapore) | Projection for 2020–2029 | PM2.5 | GEOS-Chem model for monthly emission (0.5º x 0.67º) | Long term: Jul–Oct in 2020–2029 | Modelled scenarios from LULC | Adults (age >25 years);  Children (age <5 years) | Long-term:  Adults,  Linear approximate,  1.03% increase in annual all-cause mortality per 1 μg/m^3^ increment in annual average PM2.5 concentration (Vodonos et al. 2018)[19];  Under-5 children,  Linear, 1.2% increase in annual acute lower respiratory infection (ALRI) per 1 μg/m^3^ increment in annual average PM2.5 between 0-50 μg/m^3^ and log-linear at PM2.5 >50 μg/m^3^ (Mehta et al. 2013)[20] | Mortality (all-cause mortality for adults; ALRI for children) | “Business as usual” scenario: Annual average 36,000 excess all-cause mortality in adults, and 1,100 ALRI mortality in under-5 children  “Protecting peatland” scenario: 24,000 excess mortality would be preventable | NA |
| Uda et al. (2019)[21] | (Central Kalimantan) Indonesia | 2011–2015 | PM2.5 | Hybrid Single-Particle Lagrangian Integrated Trajectory (HYSPLIT version 4.9) model | Long-term: 2011–2015 | Increment in annual average PM2.5 concentration during 2011–2015 = 26 μg/m^3^ | All-ages;  and age-specific populations (all-cause mortality), Children ages <5 years (chronic respiratory), Adults ages >30 years (CVD and LC) | Log-linear,  $RR=\left[ \frac{\left( X+1 \right)}{{(X}_{0}+1)} \right]^{\beta}$  $for X>X_{0}$;  Where $X$ refers to the annual average PM2.5 concentration over study period; $X_{0}$ refers to the lowest observed annual average PM2.5 concentration; $\beta$ is the excess mortality coefficients per-unit increment in PM2.5 ($\beta$ cardiovascular, CVD = 0.1551; $\beta$ lung cancer, LC = 0.23218; $\beta$ chronic respiratory = 0.003794; $\beta$ premature mortality = 0.001829) (Ostro 2004)[9] | Mortality (all-cause and cause specific: CVD, LC, and chronic respiratory) | Annual all-ages mortality:   - 648 all-cause - 55 chronic respiratory - 266 CVD - 95 LC   Annual age-specific mortality   - 6 chronic respiratory (ages <5) - 119 CVD (ages >30) - 42 LC (ages >30) | Linear exposure, $RR=exp\left[ \beta\left( X-X_{0} \right) \right]$;  10 μg/m^3^ changes in PM2.5 |
| Bruni Zani et al. (2020)[22] | Maritime Southeast Asia | 2005–2015 | PM2.5 | WRF-Chem | Long-term:  (Sep and Oct were defined as fire-months, while Nov was defined as non-fire months) | For both fire and non-fire years, PM10 concentration were 50.90(±23.93) μg/m^3^ in Jun–Aug, and 45.63(±15.71) μg/m^3^ in Dec-Feb.  Peak PM10 concentration in 2006 and 2015 (large fires years) >50 μg/m^3^ (reached 90 μg/m^3^) | Whole population | Global Exposure Mortality Model (GEMM)  (Specific diseases: COPD, IHD, LC, and Stroke) (Burnett et al. 2018)[23] | Mortality (Cause specific: COPD, IHD, LC, and Stroke) | Annual mortality: 150,000 (108,000–193,000) in 2005, and 204,000 (145,000–260,000) in 2015. | NA |
| Kiely et al. (2020)[24] | Maritime Southeast Asia | 2004–2015; 6 dry seasons in 2004–2015 (Aug–Oct in 2004, 2006, 2009, 2012, 2014, and 2015) | PM2.5 | WRF-Chem | Long-term: Annual average PM2.5 |  | Whole population | GEMM (Non-communicable disease and lower respiratory infections, NCD+LRI) (Burnett et al. 2018)[23] | Mortality (all-cause and LRI) | Annual mortality in 2015 = 44,041;  Total mortality for 6 high fire years = 113,600;  Total mortality in all years = 131,700 | 1% increase for every 1 μg/m^3^ increment in annual mean PM2.5 below 50 μg/m^3^ (Koplitz et al. 2016)[14] |
| Kiely et al. (2021)[25] | Indonesia | (Aug–Oct in 2004, 2006, 2009, 2012, 2014, and 2015) | PM2.5 | WRF-Chem | Long-term | In 2015, 28% average PM2.5 (from 76　μg/m^3^ to 55　μg/m^3^), and 26% population-weighted PM2.5 (from 27　μg/m^3^ to 20　μg/m^3^) were reduced due to peatland restoration. | Whole population | GEMM (NCD+LRI) (Burnett et al. 2018)[23] | Mortality; DALY  (all-cause and LRI) | In 2015:  11,914 (21%, from 55,819 to 43,905) excess mortality was reduced.  0.46 million DALY (from 2.19 million to 1.72 million) was reduced. | NA |
| Punsompong et al. (2021)[26] | Thailand | 2016 | PM2.5 | Ground-based measurement data from monitoring stations | Long-term | Annually, the Central and Northeast regions (about 59% of total populations) were exposed to PM2.5 concentrations 26–40μg/m^3^. Those in the North region (about 20% of total population) were exposed to PM2.5 >40μg/m^3^. | Whole population | IER (Specific diseases; COPD, IHD, LC, and Stroke) (Burnett et al. 2014; Cui et al. 2017)[13,27]  Threshold values:  COPD (7.17μg/m^3^,  IHD (6.96μg/m^3^),  LC (7.24μg/m^3^), Stroke (8.38μg/m^3^)  (Cui et al. 2017)[27] | Mortality  (Cause specific: COPD, IHD, LC, and Stroke) | 18,003 total premature mortality (53% stroke; 30% IHD, 12% LC, and 5% COPD) | NA |
| Reddington et al. (2021)[28] | Southern Asia (Mainland Southeast Asia, and Southeastern China) | 2003–2015 | PM2.5, Ozone | WRF-Chem | Long-term | Eliminating fire emissions would contribute to 7% reduction in population-weighted PM2.5 exposures in Southeast Asia. | Adults (age >25 years) | For PM2.5: GEMM (Specific disease: NCD+LRI) (Burnett et al. 2018)[23]  For ozone: HR = 1.14 (95% CI: 1.08, 1.21) for COPD (Turner et al. 2016)[29] | Mortality; DALY  (all-cause and LRI) | Annual PM2.5 mortality: 27,500 (24,700–30,400) per year.  Annual PM2.5 DALY: 1,047,500 (867,500–1,247,300).  Annual ozone mortality: 2,250 (2,000–2,470) per year. | NA |

PM2.5: particulate matter with aerodynamic diameter below 2.5μm; WHO: World Health Organization; LULC: land use and land cover; PSI: pollutant standard index; IER: Integrated Exposure Response; GEMM: Global Exposure Mortality Model; NCD: non-communicable disease; LRI: lower respiratory infection; CVD: cardiovascular; LC: lung cancer; COPD: chronic obstructive pulmonary disorder; IHD: ischemic heart disease; RR: relative risk; DALY: disability-adjusted life years.

**References:**

1. Johnston FH, Henderson SB, Chen Y, Randerson JT, Marlier M, DeFries RS, et al. Estimated global mortality attributable to smoke from landscape fires. Environ Health Perspect. 2012;120: 695–701. doi:10.1289/ehp.1104422

2. Morgan G, Sheppeard V, Khalaj B, Ayyar A, Lincoln D, Jalaludin B, et al. Effects of bushfire smoke on daily mortality and hospital admissions in Sydney, Australia. Epidemiology. 2010;21: 47–55. doi:10.1097/EDE.0b013e3181c15d5a

3. Sastry N. Forest fires, air pollution, and mortality in Southeast Asia. Demography. 2002. pp. 1–23. doi:10.2307/3088361

4. Hänninen OO, Salonen RO, Koistinen K, Lanki T, Barregard L, Jantunen M. Population exposure to fine particles and estimated excess mortality in Finland from an East European wildfire episode. J Expo Sci Environ Epidemiol. 2009;19: 414–422. doi:10.1038/jes.2008.31

5. Pope CA, Thun MJ, Namboodiri MM, Dockery DW, Evans JS, Speizer FE, et al. Particulate air pollution as a predictor of mortality in a prospective study of U.S. Adults. Am J Respir Crit Care Med. 1995;151: 669–674. doi:10.1164/ajrccm.151.3.7881654

6. Marlier ME, Defries RS, Voulgarakis A, Kinney PL, Randerson JT, Shindell DT, et al. El Niño and health risks from landscape fire emissions in Southeast Asia. Nat Clim Chang. 2013;3: 131–136. doi:10.1038/nclimate1658

7. Pope CA, Burnett RT, Turner MC, Cohen A, Krewski D, Jerrett M, et al. Lung cancer and cardiovascular disease mortality associated with ambient air pollution and cigarette smoke: Shape of the exposure-response relationships. Environ Health Perspect. 2011;119: 1616–1621. doi:10.1289/ehp.1103639

8. Bell ML, Dominici F, Samet JM. A meta-analysis of time-series studies of ozone and mortality with comparison to the national morbidity, mortality, and air pollution study. Epidemiology. 2005;16: 436–445.

9. Ostro B, Prüss-üstün A, Campbell-lendrum D, Corvalán C, Woodward A. Outdoor air pollution: Assessing the environmental burden of disease at national and local levels. World Heal Organ Prot Hum Environ Geneva. 2004.

10. Krewski D, Jerrett M, Burnett RT, Ma R, Hughes E, Shi Y, et al. Extended follow-up and spatial analysis of the American Cancer Society study linking particulate air pollution and mortality. HEI Research Report, 140. Health Effects Institute: Boston; 2009.

11. Crippa P, Castruccio S, Archer-Nicholls S, Lebron GB, Kuwata M, Thota A, et al. Population exposure to hazardous air quality due to the 2015 fires in Equatorial Asia. Sci Rep. 2016;6: 1–9. doi:10.1038/srep37074

12. Atkinson RW, Kang S, Anderson HR, Mills IC, Walton HA. Epidemiological time series studies of PM2.5 and daily mortality and hospital admissions: A systematic review and meta-analysis. Thorax. 2014;69: 660–665. doi:10.1136/thoraxjnl-2013-204492

13. Burnett RT, Arden Pope C, Ezzati M, Olives C, Lim SS, Mehta S, et al. An integrated risk function for estimating the global burden of disease attributable to ambient fine particulate matter exposure. Environ Health Perspect. 2014;122: 397–403. doi:10.1289/ehp.1307049

14. Koplitz SN, Mickley LJ, Marlier ME, Buonocore JJ, Kim PS, Liu T, et al. Public health impacts of the severe haze in Equatorial Asia in September-October 2015: Demonstration of a new framework for informing fire management strategies to reduce downwind smoke exposure. Environ Res Lett. 2016;11. doi:10.1088/1748-9326/11/9/094023

15. Schwartz J, Coull B, Laden F, Ryan L. The effect of dose and timing of dose on the association between airborne particles and survival. Environ Health Perspect. 2008;116: 64–69. doi:10.1289/ehp.9955

16. Anenberg SC, Schwartz J, Shindell D, Amann M, Faluvegi G, Klimont Z, et al. Global air quality and health co-benefits of mitigating near-term climate change through methane and black carbon emission controls. Environ Health Perspect. 2012;120: 831–839. doi:10.1289/ehp.1104301

17. Lepeule J, Laden F, Dockery D, Schwartz J. Chronic exposure to fine particles and mortality: An extended follow-up of the Harvard six cities study from 1974 to 2009. Environ Health Perspect. 2012;120: 965–970. doi:10.1289/ehp.1104660

18. Marlier ME, Liu T, Yu K, Buonocore JJ, Koplitz SN, DeFries RS, et al. Fires, smoke exposure, and public health: An integrative framework to maximize health benefits from peatland restoration. GeoHealth. 2019;3: 178–189. doi:10.1029/2019GH000191

19. Vodonos A, Awad YA, Schwartz J. The concentration-response between long-term PM 2.5 exposure and mortality; A meta-regression approach. Environ Res. 2018;166: 677–689. doi:10.1016/j.envres.2018.06.021

20. Mehta S, Shin H, Burnett R, North T, Cohen AJ. Ambient particulate air pollution and acute lower respiratory infections: A systematic review and implications for estimating the global burden of disease. Air Qual Atmos Heal. 2013;6: 69–83. doi:10.1007/s11869-011-0146-3

21. Uda SK, Hein L, Atmoko D. Assessing the health impacts of peatland fires: a case study for Central Kalimantan, Indonesia. Environ Sci Pollut Res. 2019;26: 31315–31327. doi:10.1007/s11356-019-06264-x

22. Bruni Zani N, Lonati G, Mead MI, Latif MT, Crippa P. Long-term satellite-based estimates of air quality and premature mortality in Equatorial Asia through deep neural networks. Environ Res Lett. 2020;15. doi:10.1088/1748-9326/abb733

23. Burnett R, Chen H, Szyszkowicz M, Fann N, Hubbell B, Pope CA, et al. Global estimates of mortality associated with longterm exposure to outdoor fine particulate matter. Proc Natl Acad Sci U S A. 2018;115: 9592–9597. doi:10.1073/pnas.1803222115

24. Kiely L, Spracklen D V., Wiedinmyer C, Conibear L, Reddington CL, Arnold SR, et al. Air quality and health impacts of vegetation and peat fires in Equatorial Asia during 2004-2015. Environ Res Lett. 2020;15. doi:10.1088/1748-9326/ab9a6c

25. Kiely L, Spracklen D V., Arnold SR, Papargyropoulou E, Conibear L, Wiedinmyer C, et al. Assessing costs of Indonesian fires and the benefits of restoring peatland. Nat Commun. 2021;12: 7044. doi:10.1038/s41467-021-27353-x

26. Punsompong P, Pani SK, Wang SH, Bich Pham TT. Assessment of biomass-burning types and transport over Thailand and the associated health risks. Atmos Environ. 2021;247: 118176. doi:10.1016/j.atmosenv.2020.118176

27. Cui H, Minjares R, Posada F, Blumberg K, Jin L, He H, et al. Cost–benefit assessment of proposed China 6 emission standard for new light-duty vehicles. 2017; 1–14.

28. Reddington CL, Conibear L, Robinson S, Knote C, Arnold SR, Spracklen D V. Air pollution from forest and vegetation fires in Southeast Asia disproportionately impacts the poor. GeoHealth. 2021;5. doi:10.1029/2021GH000418

29. Turner MC, Jerrett M, Pope CA, Krewski D, Gapstur SM, Diver WR, et al. Long-term ozone exposure and mortality in a large prospective study. Am J Respir Crit Care Med. 2016;193: 1134–1142. doi:10.1164/rccm.201508-1633OC
