# Supplementary material for: A scoping review on the health effects of smoke haze from vegetation and peatland fires in Southeast Asia: Issues with study approaches and interpretation
Source: PLoS One. 2022 Sep 15;17(9):e0274433. doi: 10.1371/journal.pone.0274433 (PMC9477317; doi:10.1371/journal.pone.0274433)
Supplement: S4 Table — (DOCX) [file pone.0274433.s005.docx]

**S4 Table. Summary of studies using combined epidemiological and health burden estimation approaches on the health effects of smoke haze in Southeast Asia**

| **Author (Year)** | **Study area** | **Study period** | **Exposure time** | **EPI** | | | | | | **EPI 🡪 HBE** | **HBE** | | |
| --- | --- | --- | --- | --- | --- | --- | --- | --- | --- | --- | --- | --- | --- |
|  |  |  |  | **Study design** | **Data source** | **Study population** | **Health outcome** | **Exposure assessment** | **Haze definition** | **Results (Concentration-response funtion)** | **Exposure level** | **Population and baseline mortality data** | **Burden estimation** |
| Chen et al. (2021)[1] | Global (43 countries) (Southeast Asia: Thailand and Philippines) | Global (2000–2016); Thailand (2000–2008); Philippines (2006–2010) | Short-term | Time-series analysis | Multi-City Multi-Country (MCC) Collaborative Research Network | Whole population | Mortality (all-cause; cardiovascular; and respiratory) | PM2.5 (GEOS-Chem model (fire-related PM2.5 were estimated based on a biomass burning inventory adapted from Global Fire Emissions Database)) | NA | RR based on 3-days moving-average (lag 0–2 days effect) RR_all-cause_: 1.019 (95% CI: 1.016, 1.022); RR_cardiovascular_: 1.017 (95% CI: 1.012, 1.021); RR_respiratory_: 1.019 (95% CI: 1.013, 1.015) | Daily maximum fire-related PM2.5 ranged from 10 to 300 in all locations; Thailand (mean: 4.28 μg/m^3^; max: 164.30 μg/m^3^); Philippines (mean: 0.79 μg/m^3^; max: 23.30 μg/m^3^) | NA | Global (All-cause: 33,510 (95% CI: 26,204–40,763); Cardiovascular: 6,993 (5,466–8,510); Respiratory: 3,503 (2,739-4,259)). Thailand (All-cause: 4,291 (3386–5175); Cardiovascular: 809 (638–975); Respiratory: 558 (440–672)). Philippines (All-cause: 436 (341–532); Cardiovascular: 140 (110–171); Respiratory: 48 (38–59)) |
| Xue et al. (2021)[2] | Global (55 Low-income and middle-income (LMIC) countries) (Southeast Asia: Indonesia, Myanmar, Vietnam, Cambodia, Philippines, Thailand, Laos, Malaysia, Singapore, Brunei) | 2000–2014 | Long-term | Matched case-control design | Demographic and Health Surveys (DHS) | Children (age <18 years) | Mortality | PM2.5 (GEOS-Chem model (fire-related PM2.5 were estimated based on a biomass burning inventory adapted from Global Fire Emission Database)) | NA | 1 μg/m^3^ increment of fire-related PM2.5 was associated with 2.31% (95% CI: 1.50, 3.13) | Monthly mean concentration of fire-related PM2.5. Among total 4.06 μg/m^3^ (SD 7.95 μg/m^3^; IQR 0.37–3.81 μg/m^3^) (population: Among case: 4.4 μg/m^3^ (SD 8.83 μg/m^3^; IQR 0.24-4.06 μg/m^3^), Among controls: 3.88 μg/m^3^ (7.44 μg/m^3^; 0.43–3.66 μg/m^3^) | Population data were obtained from the Gridded Population of the World (version 4). Baseline mortality data for children were obtained from the Global Burden of Diseases Study. | Global: 12.9 million (95% CI: 11.4 million–14.46 million); Indonesia: 28,876 (19,121–38,371); Myanmar: 10,023 (6,418–14,130); Vietnam: 4,514 (2,827–6,396); Cambodia: 3,283 (2,374–4,342); Philippines: 2,938 (1,499–7,199); Thailand: 2,645 (1,760–3,558); Laos: 2,503 (1,703–3,237); Malaysia: 1,036 (751–1,337); Singapore: 75 (66–86); Brunei: 11 (7–16) |

PM2.5: particulate matter with an aerodynamic diameter below 2.5 μm; RR: relative risk; 95% CI: 95% confidence interval; SD: standard deviation; IQR: interquartile range.

References:

1. Chen G, Guo Y, Yue X, Tong S, Gasparrini A, Bell ML, et al. Mortality risk attributable to wildfire-related PM2·5 pollution: a global time series study in 749 locations. Lancet Planet Heal. 2021;5: e579–e587. doi:10.1016/S2542-5196(21)00200-X

2. Xue T, Geng G, Li J, Han Y, Guo Q, Kelly FJ, et al. Associations between exposure to landscape fire smoke and child mortality in low-income and middle-income countries: a matched case-control study. Lancet Planet Heal. 2021;5: e588–e598. doi:10.1016/S2542-5196(21)00153-4
