# Supplementary material for: A scoping review on the health effects of smoke haze from vegetation and peatland fires in Southeast Asia: Issues with study approaches and interpretation
Source: PLoS One. 2022 Sep 15;17(9):e0274433. doi: 10.1371/journal.pone.0274433 (PMC9477317; doi:10.1371/journal.pone.0274433)
Supplement: S5 Table — (DOCX) [file pone.0274433.s006.docx]

**S5 Table. Summary of health risk assessment studies on the health effects of smoke haze in Southeast Asia**

| **Author (Year)** | **Study area** | **Study period** | **Pollutant** | **Source apportionment** | **Exposure assessment** | | **Exposure-response assessment** | | **Risk characterization** | | **Main findings** | |
| --- | --- | --- | --- | --- | --- | --- | --- | --- | --- | --- | --- | --- |
|  |  |  |  |  | **Non-carcinogenic** | **Carcinogenic** | **Non-carcinogenic** | **Carcinogenic** | **Non-carcinogenic** | **Carcinogenic** | **Non-carcinogenic** | **Carcinogenic** |
| Omar et al. (2006)[1] | (Kuala Lumpur) Malaysia | Mar 22–Dec 12, 2001 | PM10-bound PAH | Ratio of PAH: $\frac{BeP}{(BeP+BaP)}$ |  | TEF |  | not described |  | Total BaP_eq_ |  | Total BaP_eq_ (street, KL) = 0.63ng/m^3^  Total BaP_eq_ (ambient, UM) = 0.27ng/m^3^  Total BaP_eq_ (haze) = 2.60ng/m^3^  Total BaP_eq_ (haze) exceeded twice the upper limit of European legislation (0.7-1.3ng/m^3^);  both street (KL) and ambient (UM) total BaP_eq_ were lower than the limit. |
| Betha et al. (2013)[2] | (Kalimantan) Indonesia | Sep 19–Oct 12, 2009 | Trace metal elements in PM2.5 |  | CDI | CDI | RfD | SF | $HQ=\frac{\mathrm{CDI}}{\mathrm{RfD}}$ | $ELCR=CDI\times SF$ | HQ = 3.1 | $ELCR=4.7\times{10}^{-3}$ |
| Wiriya et al. (2013)[3] | (Chiang Mai) Thailand | Apr 2010, Aug–Oct 2020, and Jan–Mar 2011 | PM10-bound PAH | Fire hotspot; Diagnostic ratio; backward trajectory HYSPLIT model; Principle component analysis; Ratio of PAH: $\frac{BeP}{(BeP+BaP)}$ |  | TEQ |  | IUR_BaP_ |  | $ELCR=TEQ\times\mathrm{IUR}_{\mathrm{BaP}}$ |  | $ELCR=5.31\times{10}^{-5}$ |
| Betha et al. (2014)[4] | Singapore | Jun 20–28, and Sep 12–Oct 2, 2013 | Trace metal elements in PM2.5 | backward trajectory HYSPLIT model; Enrichment factor | CDI | CDI | RfC | SF | $HQ=\frac{\mathrm{CDI}}{\mathrm{RfD}}$ | $ELCR=CDI\times SF$ | HQ < 1 | ELCR (HRE-I) = $12\times{10}^{-6}$  ELCR (HRE-II) = $18\times{10}^{-6}$ |
| Pongpiachan et al. (2015)[5] | (9 provinces in upper northern region) Thailand | Nov 2012–Mar 2013 | PM2.5-bound PAH | Diagnostic ratio; Principal component analysis |  | BeP_eq_ |  | IUR_BaP_ |  | $\mathrm{ELCR}_{\mathrm{adults}}=BaPeq\times IURBaP$  $\mathrm{ELCR}_{\mathrm{children}}=\sum((\mathrm{BaP}_{\mathrm{eq}}\times IR\times EF\times SF\times AF)$/BW)$\times CF$  CF = 10^-6^ |  | $\mathrm{ELCR}_{\mathrm{adults}}=6.1\times{10}^{-8} \left( N-II CalEPA \right)and 6.0\times{10}^{-6} (N-I WHO)$ |
| Huang et al. (2016)[6] | Singapore | Jan–Sep 2014 | Trace metal elements in PM2.5 | Enrichment factor | CDI | CDI | RfC | SF | $HQ=\frac{\mathrm{CDI}}{\mathrm{RfD}}$ | $ELCR=CDI\times SF$ | HQ < 1 | $\mathrm{ELCR}_{sce-1}=3.41\times{10}^{-5}$  $\mathrm{ELCR}_{sce-2}=7.41\times{10}^{-5}$  $\mathrm{ELCR}_{sce-3}=1.03\times{10}^{-4}$  $(water soluble:$  $3.77\times{10}^{-5})$ |
| Khan et al. (2016)[7] | (Bangi, Selangor) Malaysia | Jul–Sep 2013. and Jan–Feb 2014 | PM2.5 constituents | Fire hotspot; Positive matrix factorization; Enrichment factor | EC_inh_ | EC_inh_ | RfC | IUR | $HQ=\frac{\mathrm{EC}_{\mathrm{inh}}}{\begin{aligned} (RfC\times\\ 1000\mu g/m^{3}) \end{aligned}}$ | $ELCR=IUR\times\mathrm{EC}_{\mathrm{inh}}$ | Highest HQ from coal burning source: $\mathrm{HQ}_{\mathrm{total}}=10.2\times{10}^{-2}$  Lowest HQ from mineral/road dust source: $\mathrm{HQ}_{\mathrm{total}}=2.3\times{10}^{-2}$ | ELCR (motor vehicle/ biomass and coal burning sources) slightly above the acceptable cancer risk level by USEPA $(1\times{10}^{-6})$  ELCR (heavy metals) = $3.9\times{10}^{-6}$ |
| Sulong et al. (2017)[8] | (Kuala Lumpur) Malaysia | Jun 2015–Jan 2016 | PM2.5 constituents | Positive matrix factorization | ADD | LADD | RfC | IUR | $HQ=\frac{\mathrm{ADD}}{\mathrm{RfC}}$  $HI=\sum\mathrm{HQ}$ | $ELCR=LADD\times IUR$ | HI (haze; non-haze) by age groups:  infant $(1.06;0.91)$ > toddler $(0.82;0.71)$ > adolescent $(0.57;0.49)$ > children $(0.53;0.45)$ > adult $(0.33;0.28)$.  HI by elements:  Cr > Mn > Cd > As > Ni | ELCR (haze; non-haze) by age groups:  adult $(2.27\times{10}^{-5};1.92\times{10}^{-5})$ > toddler $(5.50\times{10}^{-6};4.65\times{10}^{-6})$ > adolescent $(4.55\times{10}^{-6};3.84\times{10}^{-6})$ > children $(4.23\times{10}^{-6};3.58\times{10}^{-6})$ > infant $(1.42\times{10}^{-6};1.20\times{10}^{-6})$.  ELCR by elements:  Cr > As > Co > Ni > Cd > Pb |
| Urbancok et al. (2017)[9] | Singapore | May 2015–May 2016 | PM10-bound PAH | Diagnostic ratio; backward trajectory HYSPLIT model; Principal component analysis |  | LADD |  | SF |  | $ELCR=LADD\times SF$ |  | Highest ELCR (adult 18-80 years)  $ELCR=3.95\times{10}^{-7}$ |
| Sharma & Balasubramanian (2018)[10] | Singapore | 7 days in Oct 2015 | Trace metal elements in PM2.5 |  | CDI | CDI | RfD | SF | $HQ=\frac{\mathrm{CDI}}{\mathrm{RfD}}$ | $ELCR=CDI\times SF$ | HQ (light haze) = $6.99\times{10}^{-3}$  HQ (moderate haze) = $1.04\times{10}^{-2}$  HQ (severe haze) = $1.37\times{10}^{-2}$ | ELCR (light haze) = $2.82\times{10}^{-6}$  ELCR (moderate haze) = $4.39\times{10}^{-6}$  ELCR (severe haze) = $5.24\times{10}^{-6}$ |
| Sulong et al. (2019)[11] | (Kuala Lumpur) Malaysia | Jun 2015–May 2016 | PM2.5-bound PAH, LV, MN, and GL | Diagnostic ratio; Positive matrix factorization |  | LADD |  | SF |  | $ELCR=LADD\times SF$ |  | ELCR adult $(1.42\times{10}^{-7})$ >  toddler $(3.44\times{10}^{-8})$ >  adolescent $(2.84\times{10}^{-8})$ >  children $(2.64\times{10}^{-8})$ >  infant $(8.84\times{10}^{-9})$.  ELCR all = $2.40\times{10}^{-7}$ |
| Pani et al. (2020)[12] | (Chiang Mai) Thailand | Mar 19–May 11, 2016 | Black carbon | Fire hotspot; Aethalometer | EBC | EBC | N_PSC_ equivalent to a 1μg/m^3^ increase in EBC for a specific health outcome | N_PSC_ equivalent to a 1μg/m^3^ increase in EBC for a specific health outcome | N_PSC_ for CVD mortality, PLFD and LBW | N_PSC_ for lung cancer | N_PSC_ (CVD mortality) = 14.5  N_PSC_ (PLFD) = 43.5  N_PSC_ LBW = 15.3 | N_PSC_ (lung cancer) = 6.8 |
| Thepnuan et al. (2020)[13] | (Chiang Mai) Thailand | Feb 23–Apr 28, 2016 | PM2.5-bound PAH and levoglucosan | Diagnostic ratio |  | TEQ |  | not described |  | ELCR |  | $\mathrm{ELCR}_{\mathrm{BBlow}}=6.99\times{10}^{-5}$  $\mathrm{ELCR}_{\mathrm{BBmedium}}=9.74\times{10}^{-5}$  $\mathrm{ELCR}_{\mathrm{BBhigh}}=1.21\times{10}^{-4}$  $\mathrm{ELCR}_{\mathrm{BBextreme}}=1.45\times{10}^{-4}$ |
| Yabueng et al. (2020)[14] | (Chiang Mai (CM) province; Nan (NN) province) Thailand | Mar–Apr, 2017–2018 | PM2.5-bound PAH | Fire hotspot; Diagnostic ratio; backward trajectory HYSPLIT model |  | TEQ |  | IUR_BaP_ |  | $ELCR=TEQ\times$  $\mathrm{IUR}_{\mathrm{BaP}}$ |  | $\mathrm{ELCR}\left( CM station \right)={10}^{-5} \left( \mathrm{WHO} \right)\mathrm{or}$  ${10}^{-7} (CalEPA)$  $\mathrm{ELCR}\left( NN station \right)={10}^{-5\sim-4} \left( \mathrm{WHO} \right)$  $\mathrm{or}{10}^{-6} (CalEPA)$ |
| Insian et al. (2022)[15] | (Chiang Mai) Thailand | Mar–Jun, and Nov, 2019 | PM-bound PAH | Diagnostic ratio; Positive matrix factorization |  | TEQ |  | IUR_BaP_ |  | $ELCR=TEQ\times{IUR}_{BaP}$ |  | ELCR rural $(1.14\times{10}^{-4})$  ELCR urban $(6.80\times{10}^{-5})$ |

The main findings are presented for haze-related exposures; non-haze-related exposures are otherwise mentioned. The shaded area indicates inapplicable information owing to the study design. PM2.5, and PM10: particulate matter with aerodynamic diameter below 2.5μm and 10μm, respectively. PAH: polycyclic aromatic hydrocarbon; CDI: chronic daily intake; LADD: lifetime average daily dose; TEQ: toxicity equivalent concentration; RfD: reference dose; N_PSC_: Number of passively smoked cigarettes; RfC: reference concentration; SF: cancer slope factor; IUR (or IUR_BaP_): inhalation unit risk (for benzo[α]pyrene); EBC: equivalent black carbon; BaP_eq_: benzo[α]pyrene equivalent; IR: ingestion rate; EF: exposure factor; AF: adjustment factor; BW: birthweight; CF: conversion factor; EC_inh_: exposure concentration for inhalation; HQ: hazard quotient; HI: hazard index; ELCR: excess lifetime cancer risk (inclusive of other similar terminology, e.g., inhalation cancer risk (ICR)); CVD: cardiovascular; PLFD: Percentage of lung function decrement in school children; LBW: low birth weight; BBlow, BBmedium, BBhigh and BBextreme are low, medium, high, and extremely high biomass burning activities, respectively; LV: levoglucosan; MN: mannosan; GL: galactosan; Shaded cells denote non-applicable information. NPSC. HRE-I and HRE-II. sce: Scenario.

References:

1. Omar NYMJ, Mon TC, Rahman NA, Abas MR Bin. Distributions and health risks of polycyclic aromatic hydrocarbons (PAHs) in atmospheric aerosols of Kuala Lumpur, Malaysia. Sci Total Environ. 2006;369: 76–81. doi:10.1016/j.scitotenv.2006.04.032

2. Betha R, Pradani M, Lestari P, Joshi UM, Reid JS, Balasubramanian R. Chemical speciation of trace metals emitted from Indonesian peat fires for health risk assessment. Atmos Res. 2013;122: 571–578. doi:10.1016/j.atmosres.2012.05.024

3. Wiriya W, Prapamontol T, Chantara S. PM10-bound polycyclic aromatic hydrocarbons in Chiang Mai (Thailand): Seasonal variations, source identification, health risk assessment and their relationship to air-mass movement. Atmos Res. 2013;124: 109–122. doi:10.1016/j.atmosres.2012.12.014

4. Betha R, Behera SN, Balasubramanian R. 2013 Southeast Asian smoke haze: Fractionation of particulate-bound elements and associated health risk. Environ Sci Technol. 2014;48: 4327–4335. doi:10.1021/es405533d

5. Pongpiachan S, Tipmanee D, Khumsup C, Kittikoon I, Hirunyatrakul P. Assessing risks to adults and preschool children posed by PM2.5-bound polycyclic aromatic hydrocarbons (PAHs) during a biomass burning episode in Northern Thailand. Sci Total Environ. 2015;508: 435–444. doi:10.1016/j.scitotenv.2014.12.019

6. Huang X, Betha R, Tan LY, Balasubramanian R. Risk assessment of bioaccessible trace elements in smoke haze aerosols versus urban aerosols using simulated lung fluids. Atmos Environ. 2016;125: 505–511. doi:10.1016/j.atmosenv.2015.06.034

7. Khan MF, Latif MT, Saw WH, Amil N, Nadzir MSM, Sahani M, et al. Fine particulate matter in the tropical environment: monsoonal effects, source apportionment, and health risk assessment. Atmos Chem Phys. 2016;16: 597–617. doi:10.5194/acp-16-597-2016

8. Sulong NA, Latif MT, Khan MF, Amil N, Ashfold MJ, Wahab MIA, et al. Source apportionment and health risk assessment among specific age groups during haze and non-haze episodes in Kuala Lumpur, Malaysia. Sci Total Environ. 2017;601–602: 556–570. doi:10.1016/j.scitotenv.2017.05.153

9. Urbančok D, Payne AJR, Webster RD. Regional transport, source apportionment and health impact of PM10 bound polycyclic aromatic hydrocarbons in Singapore’s atmosphere. Environ Pollut. 2017;229: 984–993. doi:10.1016/j.envpol.2017.07.086

10. Sharma R, Balasubramanian R. Size-fractionated particulate matter in indoor and outdoor environments during the 2015 haze in Singapore: Potential human health risk assessment. Aerosol Air Qual Res. 2018;18: 904–917. doi:10.4209/aaqr.2017.11.0515

11. Sulong NA, Latif MT, Sahani M, Khan MF, Fadzil MF, Tahir NM, et al. Distribution, sources and potential health risks of polycyclic aromatic hydrocarbons (PAHs) in PM2.5 collected during different monsoon seasons and haze episode in Kuala Lumpur. Chemosphere. 2019;219: 1–14. doi:10.1016/j.chemosphere.2018.11.195

12. Pani SK, Wang SH, Lin NH, Chantara S, Lee C Te, Thepnuan D. Black carbon over an urban atmosphere in Northern Peninsular Southeast Asia: Characteristics, source apportionment, and associated health risks. Environ Pollut. 2020;259: 113871. doi:10.1016/j.envpol.2019.113871

13. Thepnuan D, Yabueng N, Chantara S, Prapamontol T, Tsai YI. Simultaneous determination of carcinogenic PAHs and levoglucosan bound to PM2.5 for assessment of health risk and pollution sources during a smoke haze period. Chemosphere. 2020;257. doi:10.1016/j.chemosphere.2020.127154

14. Yabueng N, Wiriya W, Chantara S. Influence of zero-burning policy and climate phenomena on ambient PM2.5 patterns and PAHs inhalation cancer risk during episodes of smoke haze in Northern Thailand. Atmos Environ. 2020;232: 117485. doi:10.1016/j.atmosenv.2020.117485

15. Insian W, Yabueng N, Wiriya W, Chantara S. Size-fractionated PM-bound PAHs in urban and rural atmospheres of northern Thailand for respiratory health risk assessment. Environ Pollut. 2022;293: 118488. doi:10.1016/j.envpol.2021.118488
