# Supplementary material for: A scoping review on the health effects of smoke haze from vegetation and peatland fires in Southeast Asia: Issues with study approaches and interpretation
Source: PLoS One. 2022 Sep 15;17(9):e0274433. doi: 10.1371/journal.pone.0274433 (PMC9477317; doi:10.1371/journal.pone.0274433)
Supplement: S1 Appendix — (DOCX) [file pone.0274433.s007.docx]

**S2 Appendix. List of articles subjected for review**

1. Brauer M, Hisham-Hashim J. Fires in Indonesia: Crisis and reaction. Environ Sci Technol. 1998;32: 13–16. doi:10.1021/es983677j
2. Aditama TY. Impact of haze from forest fire to respiratory health: Indonesian experience. Respirology. 2000;5: 169–174. doi:10.1046/j.1440-1843.2000.00246.x
3. Emmanuel SC. Impact to lung health of haze from forest fires: The Singapore experience. Respirology. 2000;5: 175–182. doi:10.1046/j.1440-1843.2000.00247.x
4. Tan WC, Qiu DW, Liam BL, Ng TP, Lee SH, van Eeden SF, et al. The human bone marrow response to acute air pollution caused by forest fires. Am J Respir Crit Care Med. 2000;161: 1213–1217. doi:10.1164/ajrccm.161.4.9904084
5. Odihi JO. Haze and Health in Brunei Darussalam: The Case of the 1997‐98 Episodes. Singap J Trop Geogr. 2001;22: 38–51. doi:10.1111/1467-9493.00092
6. Kunii O, Kanagawa S, Ismail ITS, Kunii O, Yajima I, Hisamatsu Y, et al. The 1997 haze disaster in indonesia: Its air quality and health effects. Arch Environ Health. 2002;57: 16–22. doi:10.1080/00039890209602912
7. Sastry N. Forest fires, air pollution, and mortality in Southeast Asia. Demography. 2002. pp. 1–23. doi:10.2307/3088361
8. Anaman KA, Ibrahim N. Statistical estimation of dose-response functions of respiratory diseases and societal costs of haze-related air pollution in Brunei Darussalam. Pure Appl Geophys. 2003;160: 279–293. doi:10.1007/s00024-003-8778-3
9. Frankenberg E, McKee D, Thomas D. Health consequences of forest fires in Indonesia. Demography. 2005;42: 109–129. doi:10.1353/dem.2005.0004
10. Mott JA, Mannino DM, Alverson CJ, Kiyu A, Hashim J, Lee T, et al. Cardiorespiratory hospitalizations associated with smoke exposure during the 1997 Southeast Asian forest fires. Int J Hyg Environ Health. 2005;208: 75–85. doi:10.1016/j.ijheh.2005.01.018
11. Jayachandran S. Air quality and early-life mortality: Evidence from Indonesia’s wildfires. J Hum Resour. 2009;44: 916–954. doi:10.1353/jhr.2009.0001
12. Wiwatanadate P, Liwsrisakun C. Acute effects of air pollution on peak expiratory flow rates and symptoms among asthmatic patients in Chiang Mai, Thailand. Int J Hyg Environ Health. 2011;214: 251–257. doi:10.1016/j.ijheh.2011.03.003
13. Ho RC, Zhang MW, Ho CS, Pan F, Lu Y, Sharma VK. Impact of 2013 South Asian haze crisis: Study of physical and psychological symptoms and perceived dangerousness of pollution level. BMC Psychiatry. 2014;14: 81. doi:10.1186/1471-244X-14-81
14. Othman J, Sahani M, Mahmud M, Sheikh Ahmad MK. Transboundary smoke haze pollution in Malaysia: Inpatient health impacts and economic valuation. Environ Pollut. 2014;189: 194–201. doi:10.1016/j.envpol.2014.03.010
15. Sahani M, Zainon NA, Wan Mahiyuddin WR, Latif MT, Hod R, Khan MF, et al. A case-crossover analysis of forest fire haze events and mortality in Malaysia. Atmos Environ. 2014;96: 257–265. doi:10.1016/j.atmosenv.2014.07.043
16. Yeo B, Liew CF, Oon HH. Clinical experience and impact of a community-led volunteer atmospheric haze clinic in Singapore. Southeast Asian J Trop Med Public Health. 2014;45: 1448–53. Available: http://www.ncbi.nlm.nih.gov/pubmed/26466431
17. Pothirat C, Tosukhowong A, Chaiwong W, Liwsrisakun C, Inchai J. Effects of seasonal smog on asthma and COPD exacerbations requiring emergency visits in Chiang Mai, Thailand. Asian Pacific J Allergy Immunol. 2016;34: 284–289. doi:10.12932/AP0668
18. Hassan A, Latif MT, Soo CI, Faisal AH, Roslina AM, Andrea YLB, et al. Short communication: Diagnosis of lung cancer increases during the annual southeast Asian haze periods. Lung Cancer. 2017;113: 1–3.
19. Kim Y, Knowles S, Manley J, Radoias V. Long-run health consequences of air pollution: Evidence from Indonesia’s forest fires of 1997. Econ Hum Biol. 2017;26: 186–198. doi:10.1016/j.ehb.2017.03.006
20. Sheldon TL, Sankaran C. The impact of Indonesian forest fires on Singaporean pollution and health. Am Econ Rev. 2017;107: 526–529. doi:10.1257/aer.p20171134
21. Syam AF, Elina A, Hapsari FCP, Rahardja C, Makmun D. Relation between exposure of rainforest fire smoke and clinical complaints during Indonesia rainforest fire in September-October 2015. Adv Sci Lett. 2017;23: 6739–6742. doi:10.1166/asl.2017.9385
22. Ho AFW, Wah W, Earnest A, Ng YY, Xie Z, Shahidah N, et al. Health impacts of the Southeast Asian haze problem – A time-stratified case crossover study of the relationship between ambient air pollution and sudden cardiac deaths in Singapore. Int J Cardiol. 2018;271: 352–358. doi:10.1016/j.ijcard.2018.04.070
23. Ho AFW, Zheng H, De Silva DA, Wah W, Earnest A, Pang YH, et al. The relationship between ambient air pollution and acute ischemic stroke: A time-stratified case-crossover study in a city-state with seasonal exposure to the Southeast Asian haze problem. Ann Emerg Med. 2018;72: 591–601. doi:10.1016/j.annemergmed.2018.06.037
24. Ming CR, Ban Yu-Lin A, Abdul Hamid MF, Latif MT, Mohammad N, Hassan T. Annual Southeast Asia haze increases respiratory admissions: A 2-year large single institution experience. Respirology. 2018;23: 914–920. doi:10.1111/resp.13325
25. Ho AFW, Zheng H, Earnest A, Cheong KH, Pek PP, Seok JY, et al. Time-stratified case crossover study of the association of outdoor ambient air pollution with the risk of acute myocardial infarction in the context of seasonal exposure to the Southeast Asian haze problem. J Am Heart Assoc. 2019;8: e011272. doi:10.1161/JAHA.118.011272
26. Pothirat C, Chaiwong W, Liwsrisakun C, Bumroongkit C, Deesomchok A, Theerakittikul T, et al. Influence of particulate matter during seasonal smog on quality of life and lung function in patients with chronic obstructive pulmonary disease. Int J Environ Res Public Health. 2019;16: 106. doi:10.3390/ijerph16010106
27. Suyanto S, Geater A, Chongsuvivatwong V. The effect of treatment during a haze/post-haze year on subsequent respiratory morbidity status among successful treatment tuberculosis cases. Int J Environ Res Public Health. 2019;16: 4669. doi:10.3390/ijerph16234669
28. Tan-Soo JS, Pattanayak SK. Seeking natural capital projects: Forest fires, haze, and early-life exposure in Indonesia. Proc Natl Acad Sci U S A. 2019;116: 5239–5245. doi:10.1073/pnas.1802876116
29. Aik J, Chua R, Jamali N, Chee E. The burden of acute conjunctivitis attributable to ambient particulate matter pollution in Singapore and its exacerbation during South-East Asian haze episodes. Sci Total Environ. 2020;740: 140129. doi:10.1016/j.scitotenv.2020.140129
30. Ho AFW, Zheng H, Cheong KH, En WL. The relationship between air pollution and all-cause mortality in Singapore. Atmosphere (Basel). 2020;11: 9.
31. Mueller W, Loh M, Vardoulakis S, Johnston HJ, Steinle S, Precha N, et al. Ambient particulate matter and biomass burning: an ecological time series study of respiratory and cardiovascular hospital visits in northern Thailand. Environ Heal. 2020;19: 77. doi:10.1186/s12940-020-00629-3
32. Ontawong A, Saokaew S, Jamroendararasame B, Duangjai A. Impact of long-term exposure wildfire smog on respiratory health outcomes. Expert Rev Respir Med. 2020;14: 527–531. doi:10.1080/17476348.2020.1740089
33. Vajanapoom N, Kooncumchoo P, Thach TQ. Acute effects of air pollution on all-cause mortality: A natural experiment from haze control measures in Chiang Mai Province, Thailand. PeerJ. 2020;2020: 1–15. doi:10.7717/peerj.9207
34. Zaini J, Susanto AD, Samoedro E, Bionika VC, Antariksa B. Health consequences of thick forest fire smoke to healthy residents in Riau, Indonesia: A cross-sectional study. Med J Indones. 2020;29: 58–63. doi:10.13181/mji.oa.204321
35. Jaafar H, Azzeri A, Isahak M, Dahlui M. The impact of haze on healthcare utilizations for acute respiratory diseases: Evidence from Malaysia. Front Ecol Evol. 2021;9: 764300. doi:10.3389/fevo.2021.764300
36. Mueller W, Tantrakarnapa K, Johnston HJ, Loh M, Steinle S, Vardoulakis S, et al. Exposure to ambient particulate matter and biomass burning during pregnancy: associations with birth weight in Thailand. J Expo Sci Environ Epidemiol. 2021;31: 672–682. doi:10.1038/s41370-021-00295-8
37. Pothirat C, Chaiwong W, Liwsrisakun C, Bumroongkit C, Deesomchok A, Theerakittikul T, et al. The short-term associations of particular matters on non-accidental mortality and causes of death in Chiang Mai, Thailand: a time series analysis study between 2016-2018. Int J Environ Health Res. 2021;31: 538–547. doi:10.1080/09603123.2019.1673883
38. Uttajug A, Ueda K, Oyoshi K, Honda A, Takano H. Association between PM10 from vegetation fire events and hospital visits by children in upper northern Thailand. Sci Total Environ. 2021;764: 142923. doi:10.1016/j.scitotenv.2020.142923
39. Astuti Y, Permana I, Bayu R, Rahmawati H. Distribution pattern of children with acute respiratory infection during forest fire at Central Kalimantan Indonesia. Bangladesh J Med Sci. 2022;21: 171–174. doi:10.3329/bjms.v21i1.56345
40. Jalaludin B, Garden FL, Chrzanowska A, Haryanto B, Cowie CT, Lestari F, et al. Associations between ambient particulate air pollution and cognitive function in Indonesian children living in forest fire–prone provinces. Asia Pacific J Public Heal. 2022;34: 96–105. doi:10.1177/10105395211031735
41. Phung VLH, Ueda K, Sahani M, Seposo XT, Wan Mahiyuddin WR, Honda A, et al. Investigation of association between smoke haze and under-five mortality in Malaysia, accounting for time lag, duration and intensity. Int J Epidemiol. 2022;51: 155–165. doi:10.1093/ije/dyab100
42. Siregar S, Idiawati N, Pan WC, Yu KP. Association between satellite-based estimates of long-term PM2.5 exposure and cardiovascular disease: evidence from the Indonesian Family Life Survey. Environ Sci Pollut Res. 2022;29: 21156–21165. doi:10.1007/s11356-021-17318-4
43. Johnston FH, Henderson SB, Chen Y, Randerson JT, Marlier M, DeFries RS, et al. Estimated global mortality attributable to smoke from landscape fires. Environ Health Perspect. 2012;120: 695–701. doi:10.1289/ehp.1104422
44. Crippa P, Castruccio S, Archer-Nicholls S, Lebron GB, Kuwata M, Thota A, et al. Population exposure to hazardous air quality due to the 2015 fires in Equatorial Asia. Sci Rep. 2016;6: 1–9. doi:10.1038/srep37074
45. Marlier ME, Defries RS, Voulgarakis A, Kinney PL, Randerson JT, Shindell DT, et al. El Niño and health risks from landscape fire emissions in Southeast Asia. Nat Clim Chang. 2013;3: 131–136. doi:10.1038/nclimate1658
46. Marlier ME, Liu T, Yu K, Buonocore JJ, Koplitz SN, DeFries RS, et al. Fires, smoke exposure, and public health: An integrative framework to maximize health benefits from peatland restoration. GeoHealth. 2019;3: 178–189. doi:10.1029/2019GH000191
47. Koplitz SN, Mickley LJ, Marlier ME, Buonocore JJ, Kim PS, Liu T, et al. Public health impacts of the severe haze in Equatorial Asia in September-October 2015: Demonstration of a new framework for informing fire management strategies to reduce downwind smoke exposure. Environ Res Lett. 2016;11. doi:10.1088/1748-9326/11/9/094023
48. Uda SK, Hein L, Atmoko D. Assessing the health impacts of peatland fires: a case study for Central Kalimantan, Indonesia. Environ Sci Pollut Res. 2019;26: 31315–31327. doi:10.1007/s11356-019-06264-x
49. Bruni Zani N, Lonati G, Mead MI, Latif MT, Crippa P. Long-term satellite-based estimates of air quality and premature mortality in Equatorial Asia through deep neural networks. Environ Res Lett. 2020;15. doi:10.1088/1748-9326/abb733
50. Kiely L, Spracklen D V., Wiedinmyer C, Conibear L, Reddington CL, Arnold SR, et al. Air quality and health impacts of vegetation and peat fires in Equatorial Asia during 2004-2015. Environ Res Lett. 2020;15. doi:10.1088/1748-9326/ab9a6c
51. Kiely L, Spracklen D V., Arnold SR, Papargyropoulou E, Conibear L, Wiedinmyer C, et al. Assessing costs of Indonesian fires and the benefits of restoring peatland. Nat Commun. 2021;12: 7044. doi:10.1038/s41467-021-27353-x
52. Punsompong P, Pani SK, Wang SH, Bich Pham TT. Assessment of biomass-burning types and transport over Thailand and the associated health risks. Atmos Environ. 2021;247: 118176. doi:10.1016/j.atmosenv.2020.118176
53. Reddington CL, Conibear L, Robinson S, Knote C, Arnold SR, Spracklen D V. Air pollution from forest and vegetation fires in Southeast Asia disproportionately impacts the poor. GeoHealth. 2021;5. doi:10.1029/2021GH000418
54. Chen G, Guo Y, Yue X, Tong S, Gasparrini A, Bell ML, et al. Mortality risk attributable to wildfire-related PM2·5 pollution: a global time series study in 749 locations. Lancet Planet Heal. 2021;5: e579–e587. doi:10.1016/S2542-5196(21)00200-X
55. Xue T, Geng G, Li J, Han Y, Guo Q, Kelly FJ, et al. Associations between exposure to landscape fire smoke and child mortality in low-income and middle-income countries: a matched case-control study. Lancet Planet Heal. 2021;5: e588–e598. doi:10.1016/S2542-5196(21)00153-4
56. Omar NYMJ, Mon TC, Rahman NA, Abas MR Bin. Distributions and health risks of polycyclic aromatic hydrocarbons (PAHs) in atmospheric aerosols of Kuala Lumpur, Malaysia. Sci Total Environ. 2006;369: 76–81. doi:10.1016/j.scitotenv.2006.04.032
57. Betha R, Pradani M, Lestari P, Joshi UM, Reid JS, Balasubramanian R. Chemical speciation of trace metals emitted from Indonesian peat fires for health risk assessment. Atmos Res. 2013;122: 571–578. doi:10.1016/j.atmosres.2012.05.024
58. Wiriya W, Prapamontol T, Chantara S. PM10-bound polycyclic aromatic hydrocarbons in Chiang Mai (Thailand): Seasonal variations, source identification, health risk assessment and their relationship to air-mass movement. Atmos Res. 2013;124: 109–122. doi:10.1016/j.atmosres.2012.12.014
59. Betha R, Behera SN, Balasubramanian R. 2013 Southeast Asian smoke haze: Fractionation of particulate-bound elements and associated health risk. Environ Sci Technol. 2014;48: 4327–4335. doi:10.1021/es405533d
60. Pongpiachan S, Tipmanee D, Khumsup C, Kittikoon I, Hirunyatrakul P. Assessing risks to adults and preschool children posed by PM2.5-bound polycyclic aromatic hydrocarbons (PAHs) during a biomass burning episode in Northern Thailand. Sci Total Environ. 2015;508: 435–444. doi:10.1016/j.scitotenv.2014.12.019
61. Huang X, Betha R, Tan LY, Balasubramanian R. Risk assessment of bioaccessible trace elements in smoke haze aerosols versus urban aerosols using simulated lung fluids. Atmos Environ. 2016;125: 505–511. doi:10.1016/j.atmosenv.2015.06.034
62. Khan MF, Latif MT, Saw WH, Amil N, Nadzir MSM, Sahani M, et al. Fine particulate matter in the tropical environment: monsoonal effects, source apportionment, and health risk assessment. Atmos Chem Phys. 2016;16: 597–617. doi:10.5194/acp-16-597-2016
63. Sulong NA, Latif MT, Khan MF, Amil N, Ashfold MJ, Wahab MIA, et al. Source apportionment and health risk assessment among specific age groups during haze and non-haze episodes in Kuala Lumpur, Malaysia. Sci Total Environ. 2017;601–602: 556–570. doi:10.1016/j.scitotenv.2017.05.153
64. Urbančok D, Payne AJR, Webster RD. Regional transport, source apportionment and health impact of PM10 bound polycyclic aromatic hydrocarbons in Singapore’s atmosphere. Environ Pollut. 2017;229: 984–993. doi:10.1016/j.envpol.2017.07.086
65. Sharma R, Balasubramanian R. Size-fractionated particulate matter in indoor and outdoor environments during the 2015 haze in Singapore: Potential human health risk assessment. Aerosol Air Qual Res. 2018;18: 904–917. doi:10.4209/aaqr.2017.11.0515
66. Sulong NA, Latif MT, Sahani M, Khan MF, Fadzil MF, Tahir NM, et al. Distribution, sources and potential health risks of polycyclic aromatic hydrocarbons (PAHs) in PM2.5 collected during different monsoon seasons and haze episode in Kuala Lumpur. Chemosphere. 2019;219: 1–14. doi:10.1016/j.chemosphere.2018.11.195
67. Pani SK, Wang SH, Lin NH, Chantara S, Lee C Te, Thepnuan D. Black carbon over an urban atmosphere in Northern Peninsular Southeast Asia: Characteristics, source apportionment, and associated health risks. Environ Pollut. 2020;259: 113871. doi:10.1016/j.envpol.2019.113871
68. Thepnuan D, Yabueng N, Chantara S, Prapamontol T, Tsai YI. Simultaneous determination of carcinogenic PAHs and levoglucosan bound to PM2.5 for assessment of health risk and pollution sources during a smoke haze period. Chemosphere. 2020;257. doi:10.1016/j.chemosphere.2020.127154
69. Yabueng N, Wiriya W, Chantara S. Influence of zero-burning policy and climate phenomena on ambient PM2.5 patterns and PAHs inhalation cancer risk during episodes of smoke haze in Northern Thailand. Atmos Environ. 2020;232: 117485. doi:10.1016/j.atmosenv.2020.117485
70. Insian W, Yabueng N, Wiriya W, Chantara S. Size-fractionated PM-bound PAHs in urban and rural atmospheres of northern Thailand for respiratory health risk assessment. Environ Pollut. 2022;293: 118488. doi:10.1016/j.envpol.2021.118488
