# Supplementary figures and images for: A scoping review on the health effects of smoke haze from vegetation and peatland fires in Southeast Asia: Issues with study approaches and interpretation
Source: PLoS One. 2022 Sep 15;17(9):e0274433. doi: 10.1371/journal.pone.0274433 (PMC9477317; doi:10.1371/journal.pone.0274433)

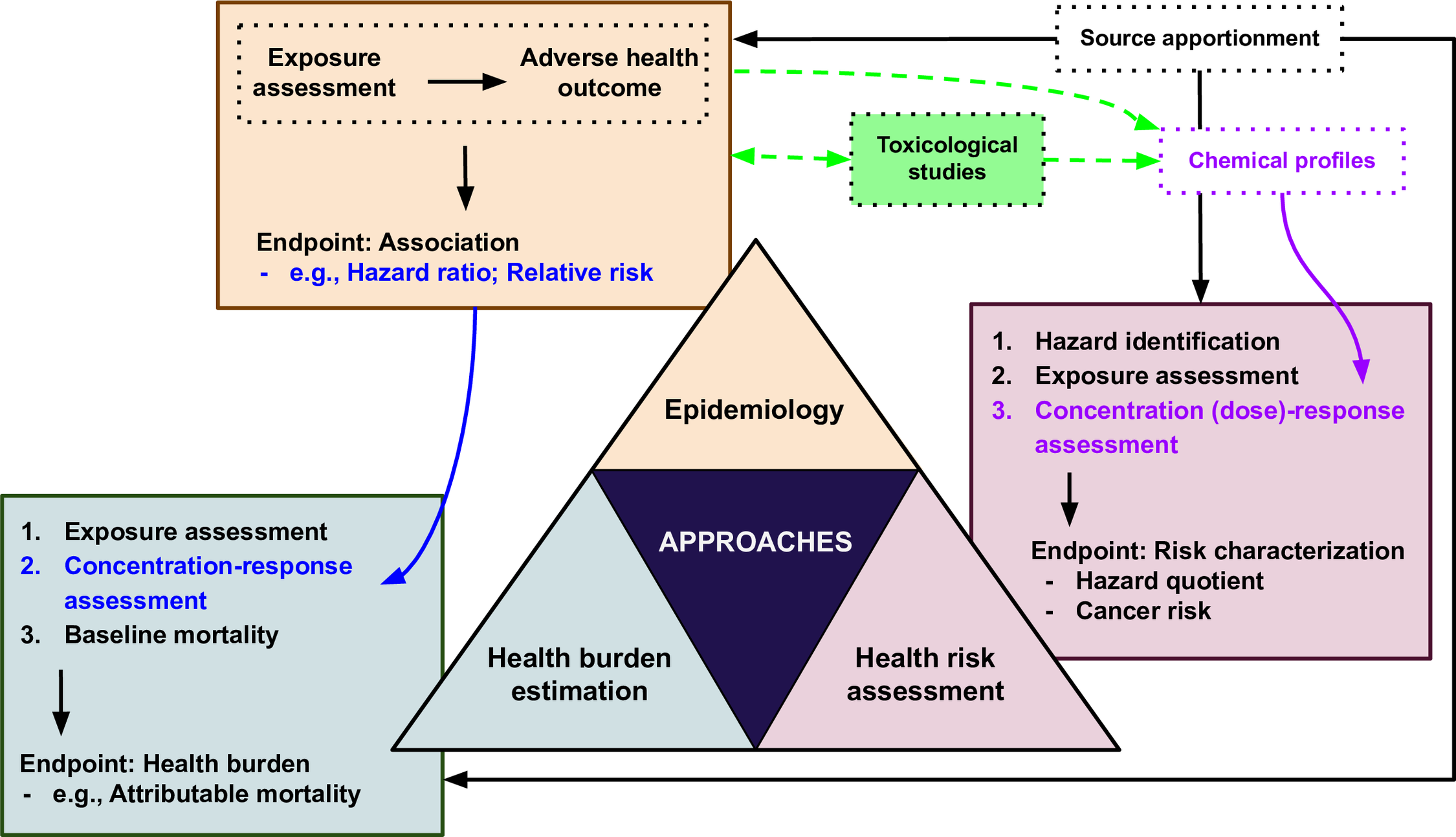

Supplement: S1 Graphical abstract — (TIF) [file pone.0274433.s008.tif]
